# Regulation of heterologous subtilin production in *Bacillus subtilis* W168

**DOI:** 10.1186/s12934-022-01782-9

**Published:** 2022-04-07

**Authors:** Qian Zhang, Carolin M. Kobras, Susanne Gebhard, Thorsten Mascher, Diana Wolf

**Affiliations:** 1 Institute of Microbiology, Technische Universität Dresden, 01217 Dresden, Germany; 2 Department Biology I, Ludwig-Maximilians-Universität München, 82152 Planegg-Martinsried, Germany; 3 Present Address: School of Biosciences, The University of Sheffield, Sheffield, S10 2TN UK; 4 Present Address: Department of Biology & Biochemistry, Milner Centre for Evolution, University of Bath, Bath, BA2 7AY UK

**Keywords:** Lantibiotic subtilin, Heterologous expression, *Bacillus subtilis* W168, *spa*-locus, Biosynthesis regulation, Two-component system (TCS), Transition state regulator AbrB

## Abstract

**Background:**

Subtilin is a peptide antibiotic (lantibiotic) natively produced by *Bacillus subtilis* ATCC6633. It is encoded in a gene cluster *spaBTCSIFEGRK* (*spa*-locus) consisting of four transcriptional units: *spaS* (subtilin pre-peptide), *spaBTC* (modification and export), *spaIFEG* (immunity) and *spaRK* (regulation). Despite the pioneer understanding on subtilin biosynthesis, a robust platform to facilitate subtilin research and improve subtilin production is still a poorly explored spot.

**Results:**

In this work, the intact *spa*-locus was successfully integrated into the chromosome of *Bacillus subtilis* W168, which is the by far best-characterized Gram-positive model organism with powerful genetics and many advantages in industrial use. Through systematic analysis of *spa*-promoter activities in *B. subtilis* W168 wild type and mutant strains, our work demonstrates that subtilin is basally expressed in *B. subtilis* W168, and the transition state regulator AbrB strongly represses subtilin biosynthesis in a growth phase-dependent manner. The deletion of AbrB remarkably enhanced subtilin gene expression, resulting in comparable yield of bioactive subtilin production as for *B. subtilis* ATCC6633. However, while in *B. subtilis* ATCC6633 AbrB regulates subtilin gene expression via SigH, which in turn activates *spaRK,* AbrB of *B. subtilis* W168 controls subtilin gene expression in SigH-independent manner, except for the regulation of *spaBTC*. Furthermore, the work shows that subtilin biosynthesis in *B. subtilis* W168 is regulated by the two-component regulatory system SpaRK and strictly relies on subtilin itself as inducer to fulfill the autoregulatory circuit. In addition, by incorporating the subtilin-producing system (*spa*-locus) and subtilin-reporting system (P_*psdA*_-*lux*) together, we developed “online” reporter strains to efficiently monitor the dynamics of subtilin biosynthesis.

**Conclusions:**

Within this study, the model organism *B. subtilis* W168 was successfully established as a novel platform for subtilin biosynthesis and the underlying regulatory mechanism was comprehensively characterized. This work will not only facilitate genetic (engineering) studies on subtilin, but also pave the way for its industrial production. More broadly, this work will shed new light on the heterologous production of other lantibiotics.

**Supplementary Information:**

The online version contains supplementary material available at 10.1186/s12934-022-01782-9.

## Introduction

The increasing threat of multidrug-resistant bacteria paired with a small number of emergency antibiotics and empty pipelines in research of new antimicrobials has prompted scientists to search for new antimicrobial agents to fight bacterial infections. In the line of research, a group of ribosomally-synthesized antimicrobial peptides (AMPs), called lantibiotics, has gained great attention as promising alternatives to classic antibiotics [1, 2]. Lantibiotics are characterized by the presence of lanthionine and methyllanthionine bridges [3, 4], and predominantly act against Gram-positive bacteria, including pathogenic organisms, such as methicillin-resistant *Staphylococcus aureus* (MRSA) and vancomycin-resistant enterococci (VRE) [5]. Many characteristics and chemical properties that lantibiotics hold, like low molecular weights, thermal and protease stability, no or very low toxicity, low tendency to generate resistance and low immunogenicity, render them suitable for potential applications in different healthcare-associated settings such as human and veterinary medicine, and biochemical, pharmaceutical, agricultural or food industries [6, 7]. The ribosomal pathway of lantibiotic biosynthesis also confers clear advantages for bioengineering (to generate variants with improved capability) over the non-ribosomally synthesized compounds because of the direct link between gene sequence and product [6, 8, 9].

Subtilin is a member of class I lantibiotics originally produced by *Bacillus subtilis* ATCC6633 and was the first lantibiotic produced by *Bacillus* strain to be described [10, 11]. It shares significant structural and functional similarities to nisin, which is the most prominent and to date the only lantibiotic that has been commercially used in food industry [2, 11]. As with nisin, subtilin shows antimicrobial activity in a nanomolar range against a broad spectrum of Gram-positive bacteria, such as *Micrococcus luteus*, *Lactococcus* spp. and *Bacillus* spp. [12, 13]. It docks on lipid II (an intermediate of cell wall biosynthesis), thus inhibiting bacterial growth, and the aggregation of subtilin-lipid II complex may also result in the permeabilization of the cytoplasmic membrane triggering cell lysis [13].

**Fig. 1:**
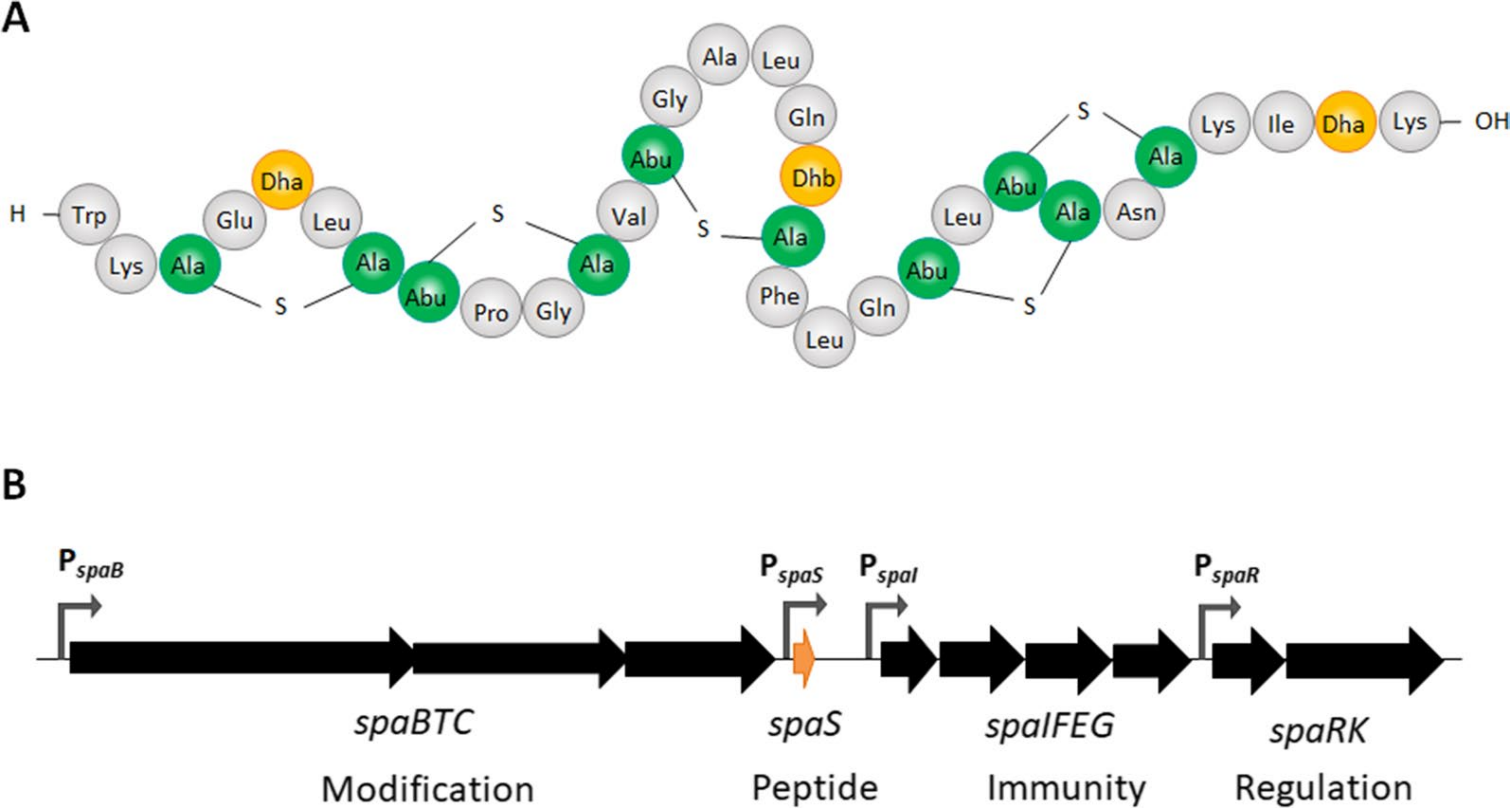
Structure of subtilin and the biosynthesis gene cluster. **A** Mature subtilin contains 32 amino acids indicated in three-letter code. The lanthionine (Ala-S-Ala) and β-methyl-lanthionine (Abu-S-Ala) bridges are in green. Dehydrated residues are in orange, with Dha represents didehydroalanine and Dhb didehydrobutyrine. **B** The *spa*-locus (*spaBTCSIFEGRK, ∼12 kb*) includes 10 genes that are transcribed in four transcriptional units. The promoters for each transcriptional unit are indicated with grey arrows. P_*spaS*_ precedes the structural gene *spaS*; P_*spaB*_ leads *spaBTC* operon which encodes proteins involved in the modification and export of subtilin prepeptide; P_*spaI*_ controls immunity genes *spaIFEG* for self-protection of subtilin-producing strain; P_*spaR*_ drives the expression of *spaRK* for two-component regulatory system SpaRK. The schematics are modified from [45] and not drawn to scale

The genes involved in subtilin biosynthesis are organized in a gene cluster *spaBTCSIFEGRK* (*spa*-locus) (Fig. 1). The structural gene *spaS* encodes the subtilin precursor, which is subject to post-translational modification by SpaBC and export to the outside of the cell by SpaT [14, 15]. On the trans side of the membrane an N-terminal leader peptide is cleaved off the subtilin precursor by some of the proteases, such as AprE and WprA, produced by *B. subtilis* ATCC6633, generating the bioactive subtilin [16, 17]. The self-protection of the producing strain is mediated by immunity genes *spaIFEG*, in which the lipoprotein SpaI acts as subtilin-intercepting protein competing with the formation of subtilin-lipid II complexes, and the SpaFEG transporter expels bioactive subtilin from cell membrane, thus diminishing cell-associated subtilin and protecting the cell [18]. Subtilin biosynthesis and immunity in *B. subtilis* ATCC6633 are under dual-control of the two-component system (TCS) SpaRK and the transition state regulator AbrB via the alternative sigma factor SigH which positively regulates *spaRK* expression. During the exponential growth phase, AbrB acts as a repressor of subtilin biosynthesis, and subtilin is basally expressed at a low level [19]. At the end of the exponential phase, AbrB synthesis is repressed, followed by the derepression of SigH and thereby activation of TCS SpaRK. At a threshold concentration of extracellular subtilin, the lantibiotic acts as autoinducing molecule and activates SpaRK, which in turn initiates the transcription of *spaIFEG*, *spaBTC* and *spaS* through the activated response regulator SpaR [20–22].

These developments are encouraging for further subtilin study, however, the native producer *B. subtilis* ATCC6633 is not tractable for genetic modification. Thus, it would be of great advantage if the subtilin gene cluster were available in a strain that is both, amenable to genetic manipulation and suitable for industrial production. With this consideration, *B. subtilis* 168 strain that is one of the best-characterized Gram-positive model strains, appeared to be the most promising platform. *B. subtilis* 168 has been the focus of diverse research interest in academic and industrial settings, benefit from the ease of genetic manipulation because of its natural competence and the wealth of available physiological and biochemical data. The rapid growth rate, well-characterized protein secretion ability, excellent fermentation properties and the lack of toxic by-products render *B. subtilis* 168 indispensable for industrial applications [23–25]. *B. subtilis* 168 was previously explored as a heterologous subtilin producer by transformation with genomic DNA from *B. subtilis* ATCC6633 [26]. Although subtilin producing transformants were isolated, the limited understanding of *spa*-locus at the time did not allow further investigation [26]. More recently, van Tilburg et al. used the genome-minimized *B. subtilis* 168 strain PG10 (Mini*Bacillus* PG10) as a host for subtilin production by introducing the structural gene *spaS* and modification genes *spaBTC*, instead of the whole *spa*-locus, into its genome [17]. However, the lack of extracellular proteases in this strain, required for the maturation of subtilin, meant that additional in vitro proteolytic degradation steps were required to produce bioactive subtilin [17].

In this study, we sought to exploit *B. subtilis* W168 (a derivative strain of *B. subtilis* 168) as a robust workhorse for heterologous subtilin production by integrating the well-characterized subtilin gene cluster *spa*-locus into its chromosome [27]. Systematic analysis using *spa* promoter-reporter (*lux*) fusions revealed the basal expression of *spa* genes in *B. subtilis* W168 and the control of subtilin biosynthesis by TCS SpaRK, which strictly relied on subtilin molecule itself for autoregulation. More importantly, we found that AbrB in *B. subtilis* W168 is a strong inhibitor of subtilin biosynthesis, and its deletion remarkably enhanced the yield of bioactive subtilin to a comparable level as in the native producer *B. subtilis* ATCC6633. Surprisingly, AbrB was found to regulate subtilin expression in a distinct manner from that found in *B. subtilis* ATCC6633 [19]. Furthermore, we also developed “online” reporters by incorporating the subtilin-producing system (*spa*-locus) and subtilin-reporting system (P_*psdA*_/P_*liaI*_-*lux*) together in *B. subtilis* W168 to monitor the dynamics of subtilin biosynthesis.

## Results

### Subtilin genes are basally expressed in *B. subtilis* W168

To get an idea of how *spa*-locus expression and thereby subtilin production will behave in *B. subtilis* W168, firstly the basal expression of the *spa* promoters P_*spaI*_, P_*spaB*_, P_*spaS*_ and P_*spaR*_ was investigated. The four promoter fragments were amplified using the genome DNA of *B. subtilis* ATCC6633 as template and the primer pairs listed in Additional file 1: Table S3. All of the putative regulatory elements within the promoters based on previous studies have been included [19, 20, 22]. The respective promoter was fused to a *luxABCDE* cassette as a reporter of promoter activity and integrated into the *sacA* locus of *B. subtilis* W168 chromosome (Additional file 1: Table S1). A promoterless P_*empty*_-*lux* fusion was used as control for background activity of the luciferase.

**Fig. 2:**
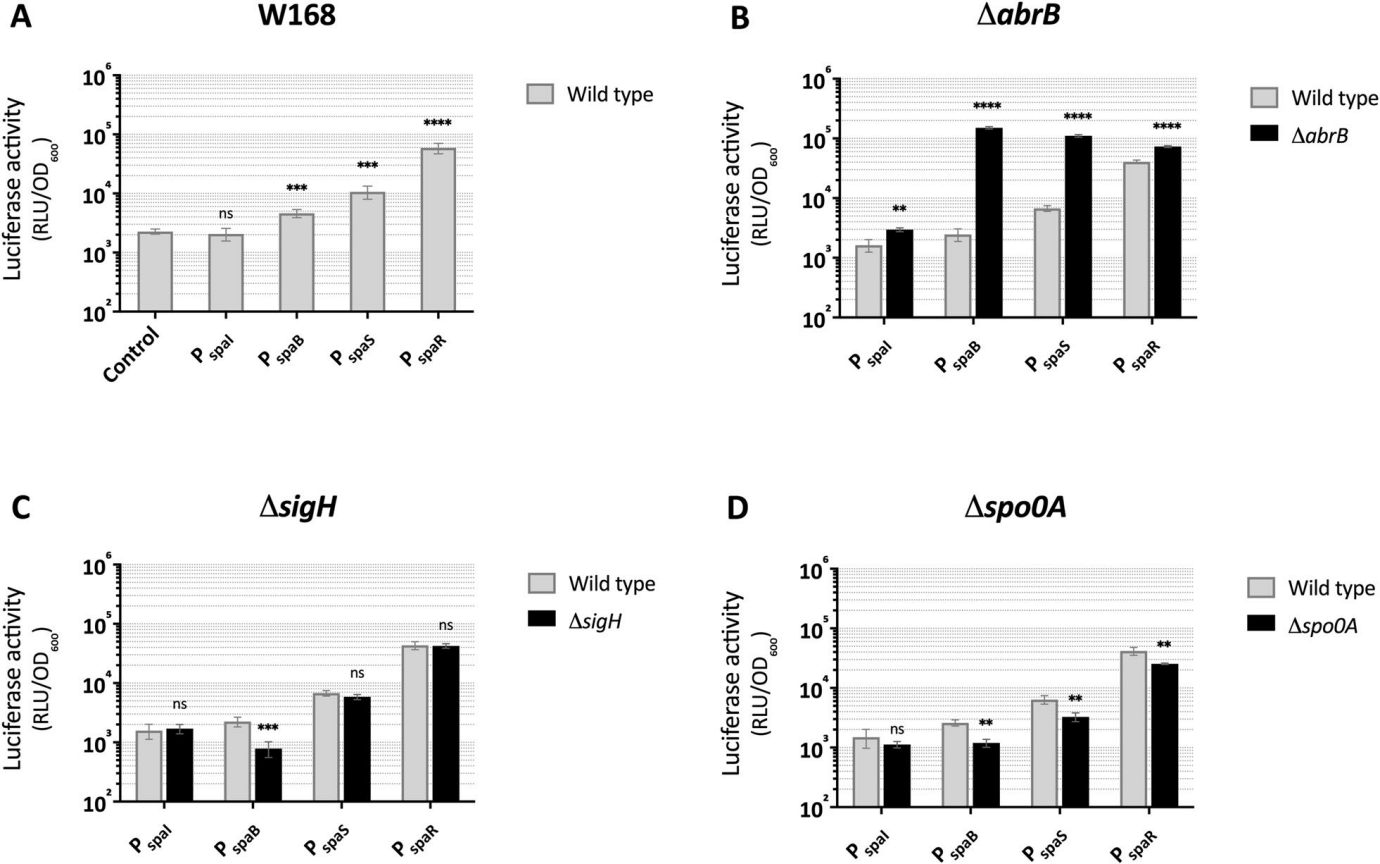
Activity of *spa* promoters in wild type *B. subtilis* W168 and single mutants. *spa* promoters fused to the luciferase cassette (*luxABCDE*) were integrated into the chromosome of *B. subtilis* W168 (**A**) and the mutants Δ*abrB* (**B**), Δ*sigH* (**C**) and Δ*spo0A* (**D**), respectively. The activity of *spa* promoters in different genetic backgrounds was evaluated using a microplate reader and represented by the luciferase activity (RLU/OD_600_), which is shown as the raw luminescence output (relative luminescence units, RLU) normalized by the cell density (OD_600_). The dynamics of *spa* promoter activity during growth are shown in Additional file 3: Fig. S2. The respective peak activity around the transition point into stationary phase was plotted here to represent the corresponding promoter activity. The strain containing a promoterless *lux* fusion was used as control as shown in **A**. Error bars represent the standard deviations from two biological replicates with each measurement carried out in triplicates. *ns* not significant, *p ≤ 0.05, **p ≤ 0.01, ***p ≤ 0.001, ****p ≤ 0.0001

Three of the promoters, P_*spaB*_, P_*spaS*_ and P_*spaR*_, displayed basal activities at different levels (Fig. 2A). P_*spaR*_ exhibited the strongest activity (26-fold) compared to the control. P_*spaS*_ and P_*spaB*_ showed weaker activity with, respectively, 4.7- and 2-fold changes compared to the control. However, no detectable basal activity was observed for P_*spaI*_ under these conditions. The differing activities of the *spa* promoters in absence of subtilin was first time reported in our study, in particular the high activity of P_*spaR*_. To examine whether the presence of *spa*-locus would affect the promoter activity in *B. subtilis* W168, the promoters were further measured in *B. subtilis* W168 carrying *spa*-locus, respectively, which revealed the virtually identical behavior of *spa* promoters in the presence and absence of *spa*-locus (Additional file 2: Fig. S1). The results suggest that subtilin biosynthesis genes can be basally expressed in *B. subtilis* W168 even when subtilin molecules are absent. The dynamics of the promoter activity along the cell growth was consistent with the findings that had been observed previously in *B. subtilis* ATCC6633, i.e., *spa* promoters were activated at mid-exponential growth phase and reached the maximum activity at the transition point into the stationary growth phase followed by gradually decreasing activity [19] (Additional file 2: Fig. S2A).

### AbrB significantly represses subtilin gene expression in *B. subtilis* W168

AbrB as a pleiotropic transition state regulator regulates many post-exponentially expressed events, such as antibiotic production and sporulation in *B. subtilis* [28]. SigH is an alternative sigma factor representing a typical regulator of late-growth activities [29, 30]. The transcription of *sigH* is under negative control of AbrB, whose synthesis in turn is repressed by Spo0A [31, 32]. In *B. subtilis* ATCC6633, subtilin biosynthesis is regulated by AbrB via SigH that activates the expression of *spaRK* [19].

To unveil the regulation of subtilin expression by AbrB and SigH in *B. subtilis* W168, *abrB* and *sigH* were individually deleted in the strains carrying the different *spa* promoter-*lux* fusions. As shown in Fig. 2B, the absence of *abrB* remarkably enhanced the activity of *spa* promoters, especially P_*spaB*_ and P_*spaS*_, showing over 60-fold and 16-fold increased activity, respectively. P_*spaI*_ and P_*spaR*_ were up-regulated at lower levels compared to P_*spaB*_ and P_*spaS*_. Notably, the effect of AbrB mutation on subtilin gene expression could be observed already in early exponential phase (Additional file 2: Fig. S2B), suggesting a significant repression of AbrB on subtilin biosynthesis during the exponential growth phase.

Unexpectedly, the deletion of SigH did not affect the activity of *spa* promoters, with the exception of P_*spaB*_ whose activity was decreased by 2.85-fold (Fig. 2C). The result indicates that, unlike its role as activator of P_*spaR*_ in *B. subtilis* ATCC6633, SigH of *B. subtilis* W168 appeared to solely regulate P_*spaB*_. In addition, inactivation of *spo0A* led to an overall decrease of *spa* promoter activity by 1.3- to 2.2-fold (Fig. 2D). Presumably, the lack of Spo0A derepressed *abrB*, which resulted in higher levels of AbrB and consequentially stronger repression of *spa* gene expression.

### SigH solely regulates the expression of *spaBTC*

**Fig. 3:**
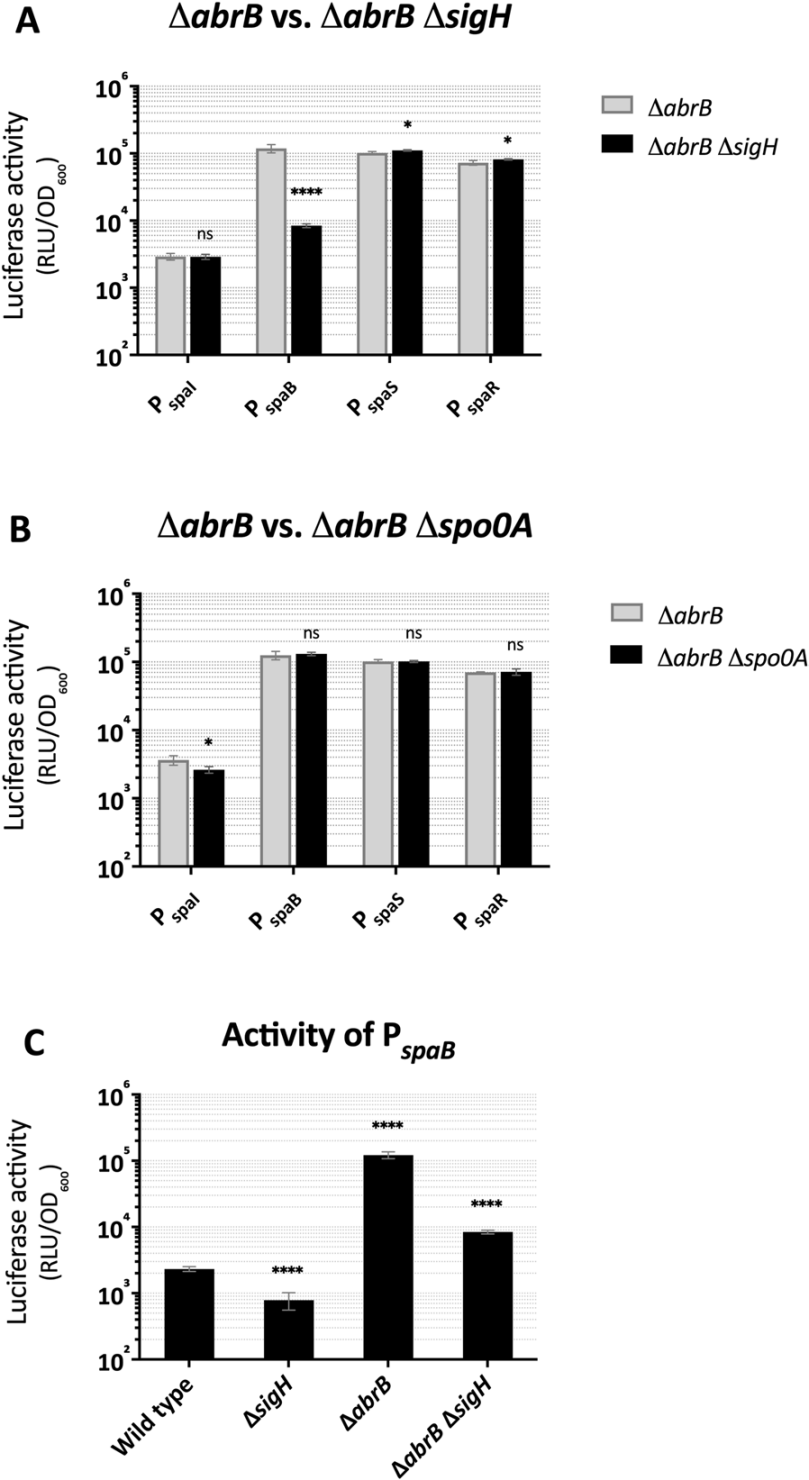
Activity of *spa* promoters in *B. subtilis* W168 double mutants. The activity of *spa* promoters in double mutants, Δ*abrB* Δ*sigH* (**A**) and Δ*abrB* Δ*spo0A* (**B**), was evaluated and presented as described for Fig. 2. The dynamics of the promoter activity in double mutants during growth are shown in Additional file 4: Fig. S3. (**C**) represents the activity of the *spaB* promoter in different *B. subtilis* W168-derived genetic backgrounds. Error bars represent the standard deviations from two biological replicates with each measurement carried out in triplicates. *ns* not significant, *p ≤ 0.05, **p ≤ 0.01, ***p ≤ 0.001,****p ≤ 0.0001

The regulatory cascade between SigH, AbrB and Spo0A is clear as mentioned above, but could it be possible that they somehow regulate subtilin gene expression independently of each other, especially with respect to their influence on P_*spaB*_? To answer this question, double mutants of Δ*abrB* Δ*sigH* and Δ*abrB* Δ*spo0A* harboring different *spa* promoter-*lux* fusions were generated. The response of the *spa* promoters in the regulator double mutation backgrounds was compared with that in the respective single mutants. As shown in Fig. 3A, P_*spaI*_, P_*spaS*_ and P_*spaR*_ displayed an identical behavior in the Δ*abrB* Δ*sigH* double mutant as in Δ*abrB* single mutant, suggesting that these three promoters are regulated by AbrB but are independent of SigH. Different from the scenario, the SigH of *B. subtilis* ATCC6633 directly and only regulates P_*spaR*_ [19]. In contrast, P_*spaB*_ again appeared to be an exception by showing obviously lower activity in Δ*abrB* Δ*sigH* double mutant than in the Δ*abrB* single mutant. Additionally, compared to that in wild type, P_*spaB*_ activity in Δ*abrB* Δ*sigH* was still elevated (Fig. 3C). The data suggest that SigH only plays a role in positively regulating P_*spaB*_ and that AbrB acts as a repressor of *sigH* in this pathway. However, this does not appear to be the only way in which AbrB regulates P_*spaB*_, otherwise P_*spaB*_ should have shown identical activity in Δ*abrB* Δ*sigH* as in Δ*sigH*. Instead, while deletion of *sigH* alone led to a marked decrease in P_spaB_ activity, additional deletion of *abrB* caused an increased activity above wild-type levels, but below those of the *abrB* single mutant (Fig. 3C). Based on the results, we therefore propose two possible pathways of regulation on P_*spaB*_: (I) the regulation by AbrB without the involvement of SigH in between and (II) the regulation by AbrB via SigH.

Furthermore, all *spa* promoters showed identical behavior in Δ*abrB* Δ*spo0A* double mutant as in the Δ*abrB* single mutant (Fig. 3B). This was not surprising, as it confirmed that Spo0A acts upstream of AbrB as a repressor of the *abrB* gene. Thus, any effect caused by *spo0A* deletion can be bypassed when *abrB* gene is deficient. Taken together, the results demonstrate that AbrB regulates subtilin gene expression independently of SigH (except for P_*spaB*_) in *B. subtilis* W168, presenting a distinct regulatory scenario from the regulation of subtilin gene expression by AbrB via SigH in *B. subtilis* ATCC6633 [19]. Moreover, SigH of *B. subtilis* W168 solely acted as a positive regulator of *spaBTC* expression.

### Autoregulation of subtilin biosynthesis via two-component system SpaRK

In addition to growth phase-dependent expression, subtilin biosynthesis is also regulated via the two-component regulatory system SpaRK in *B. subtilis* ATCC6633 [19]. Upon sensing extracellular subtilin at a critical concentration, membrane-spanning SpaK is activated and transfers the signal to the intracellular response regulator SpaR through a phosphorylation cascade. Activated SpaR subsequently binds to target promoters (P_*spaI*_, P_*spaB*_ and P_*spaS*_) and initiates gene transcription of subtilin biosynthesis and self-immunity. Subtilin production thereby generates an auto-induction loop that ensures a massive and collective response in the cell community. This process is also termed quorum-sensing, a strategy of cell-to-cell communication generally found in bacteria [21].

**Fig. 4:**
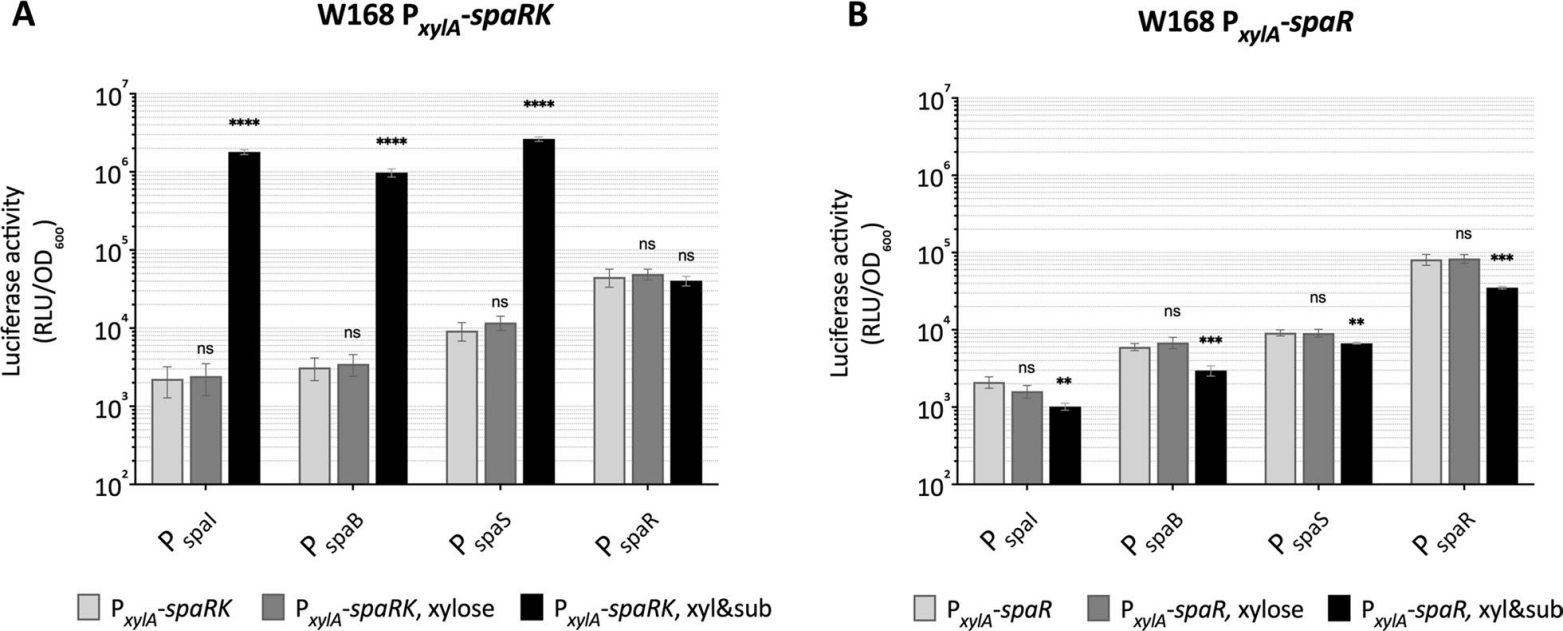
Regulation of subtilin gene expression by the TCS SpaRK. The activity of *spa* promoters in the presence of SpaRK (**A**) or SpaR (**B**) under control of a xylose-inducible promoter was evaluated and present as described for Fig. 2. The response of *spa* promoters to SpaRK or SpaR under conditions of untreated, the addition of xylose, and the addition of both xylose (0.5%, w/v) and subtilin (0.75% subtilin supernatant, v/v) as indicated as xyl&sub, is displayed. The dynamics of *spa* promoter activities during growth are shown in Additional file 5: Fig. S4. Error bars represent the standard deviations of two biological replicates with each measurement carried out in triplicates. *ns* not significant, *p ≤ 0.05, **p ≤ 0.01, ***p ≤ 0.001,****p ≤ 0.0001

To verify this auto-regulatory mechanism of subtilin biosynthesis in *B. subtilis* W168, *spaRK*, encoding the TCS SpaRK, was fused to the xylose-inducible promoter P_*xylA*_ and integrated into the genome of *B. subtilis* W168 carrying different *spa* promoters. The results show that in the absence of subtilin, overexpression of *spaRK* did not induce the *spa* promoters under any given conditions, while the addition of subtilin triggered a dramatic activation of P_*spaI*_ (800-fold), P_*spaB*_ (311-fold) and P_*spaS*_ (283-fold), but not P_*spaR*_, as shown in Fig. 4A. The data substantiate that the TCS SpaRK mediates the regulatory circuit of subtilin biosynthesis and immunity in *B. subtilis* W168 and strictly relies on subtilin as autoinducer, but SpaRK does not regulate the expression of its own encoding operon.

It has been reported that overproduction of a TCS response regulator alone can enable its activity, including the OmpR and PhoP of *E. coli* which belong to the same subfamily of response regulator as SpaR [33–38]. To investigate if SpaR alone is sufficient to regulate *spa* gene expression in *B. subtilis* W168, *spaR* was placed under control of the xylose-inducible promoter P_*xylA*_ and integrated into the genome of *B. subtilis* W168 strains carrying different *spa* promoters. However, results indicate that overexpression of SpaR alone in *B. subtilis* W168 was not sufficient to activate *spa* promoters in any given conditions (Fig. 4B). This suggests that the histidine kinase SpaK is indispensable for the regulatory function of the TCS SpaRK in subtilin biosynthesis in *B. subtilis* W168.

### Subtilin produced by *B. subtilis* W168 shows antimicrobial activity

With the understanding of the regulatory mechanism of subtilin biosynthesis, the subtilin gene cluster (*spa*-locus) from *B. subtilis* ATCC6633 strain was transferred into *B. subtilis* W168 by integration into the *amyE* locus of its chromosome. Given the strong repression of AbrB on subtilin gene expression, *abrB* was deleted in *B. subtilis* W168 carrying the *spa*-locus to examine the actual impact of AbrB on subtilin production, as well as *sigH* and *spo0A*.

**Fig. 5:**
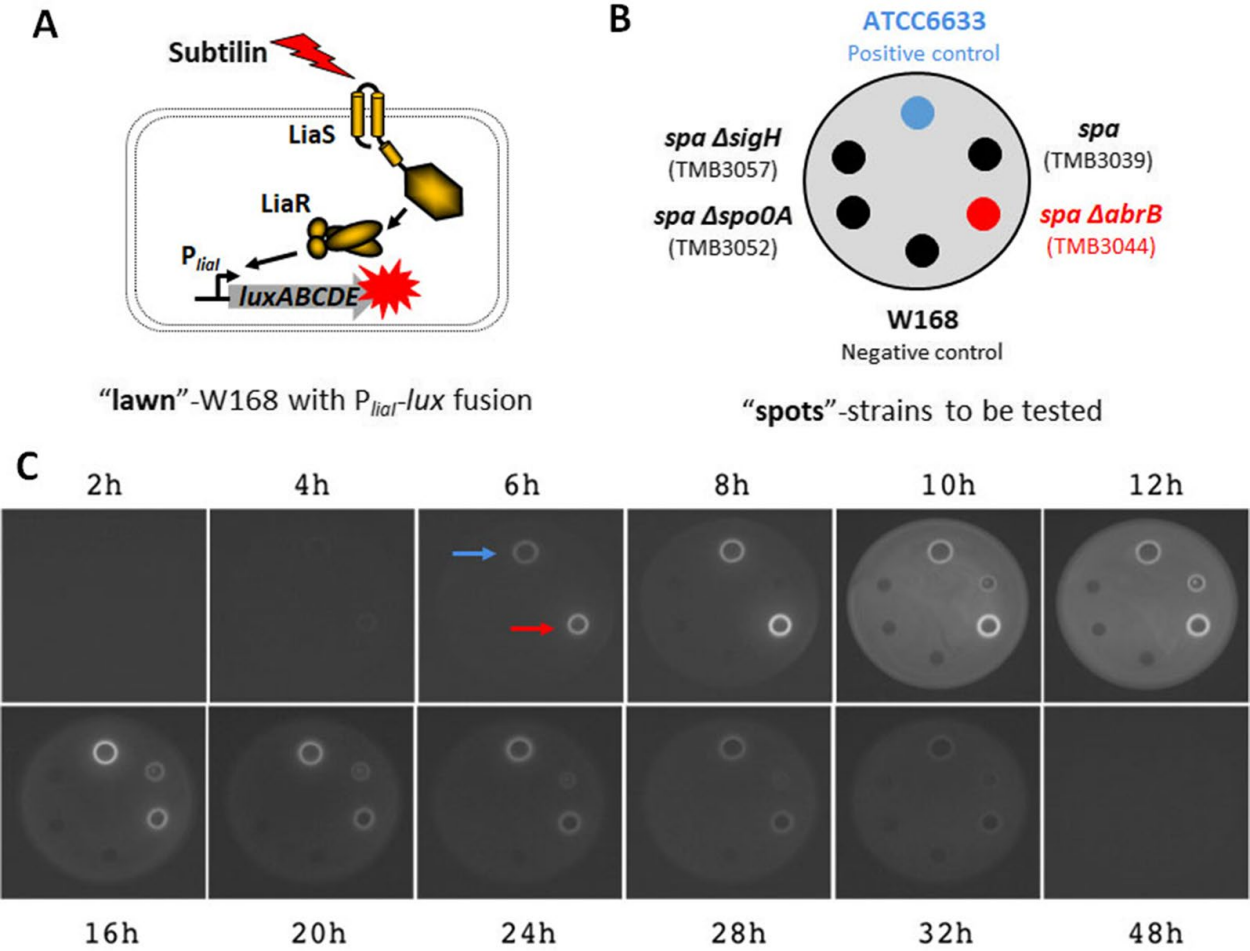
Spot-on-lawn assay revealed the production of bioactive subtilin in *B. subtilis* W168. **A** *B. subtilis* W168 P_*liaI*_-*lux* (TMB1617) strain containing the subtilin-sensitive promoter P_*liaI*_ fused to the *lux* reporter was used as a detector (“lawn”) for subtilin production. P_*liaI*_ is controlled by the TCS LiaRS encoded in the *liaIHGFSR* operon of *B. subtilis* W168. In response to subtilin, LiaRK is activated through “damage-sensing” and induces P_*liaI*_, and thus expression of the luciferase cassette (*luxABCDE*), which can be measured under a bioluminescence imaging device. **B** Application schematic of “spots” on “lawn”. **C** The time course of subtilin production by luminescence measurement. Luminescence was measured every 2 h up to 20 h and subsequently in longer intervals up to 48 h. The bright circles in contrast to the background around the “spot” position indicate the induced luminescence production by subtilin. Blue and red arrows on the plate at the 6^th^ hour mark the positive control (*B. subtilis* ATCC6633), and *B. subtilis* W168 strain carrying *spa*-locus and *abrB* mutation (TMB3044), respectively. The pictures are representatives for triplicates of measurements

“Spot-on-lawn” assays were exploited to efficiently evaluate subtilin production. The reporter strain (TMB1617, P_*liaI*_-*lux*) mixed with soft agar was grown on LB plate as “Lawn”, on which the potential subtilin-producing strains were spotted (Fig. 5B). The reporter strain contains the subtilin-sensitive promoter P_*liaI*_ fused to the *lux* cassette (Fig. 5A). In the presence of antimicrobially active subtilin, the TCS LiaRS, encoded in the *liaIHGFSR* operon of *B. subtilis* W168, is activated through a “damage-sensing” mechanism, which consequentially induces P_*liaI*_ and the expression of the reporter gene [39, 40]. Thus, the output of bioluminescence at defined time points during the growth course can be indicative of the production of bioactive subtilin.

As shown in Fig. 5C, *B. subtilis* W168 containing the *spa*-locus and *abrB* deletion (TMB3044) produced a comparable amount of active subtilin compared to the positive control (*B. subtilis* ATCC6633). However, the presence of AbrB strongly inhibited subtilin biosynthesis, as evidenced by the rather weak luminescence signal displayed by the strain TMB3039 containing the wild type *abrB* gene. No detectable bioluminescence was observed from TMB3057 (*B. subtilis* W168 *spa* Δ*sigH*) and TMB3052 (*B. subtilis* W168 *spa* Δ*spo0A*), consistent with the negative effect of *sigH* and *spo0A* deletion on subtilin expression explained above. For both TMB3044 (red arrow in Fig. 5C) and *B. subtilis* ATCC6633 (blue arrow in Fig. 5C), the luminescence signal was detected as early as the 6^th^ hour of growth. The signal intensity gradually increased to a maximum level and sustained for several hours, followed by a gradually decrease in intensity. Notably, the luminescence signal of TMB3044 reached the maximum level earlier than the signal of *B. subtilis* ATCC6633 (Fig. 5C). Please note that due to the auto-induction of P_*liaI*_ promoter at the transition point into stationary growth phase [41], the plate at the 10^th^ and 12^th^ hour of incubation showed a strong background luminescence signal elicited by the “Lawn”. Taken together, the results nicely demonstrate that *B. subtilis* W168 is compatible with the heterologous subtilin gene cluster and can produce a promising amount of bioactive subtilin when the repressor AbrB is absent.

### Quantification and the antimicrobial activity of heterologous subtilin production

**Fig. 6:**
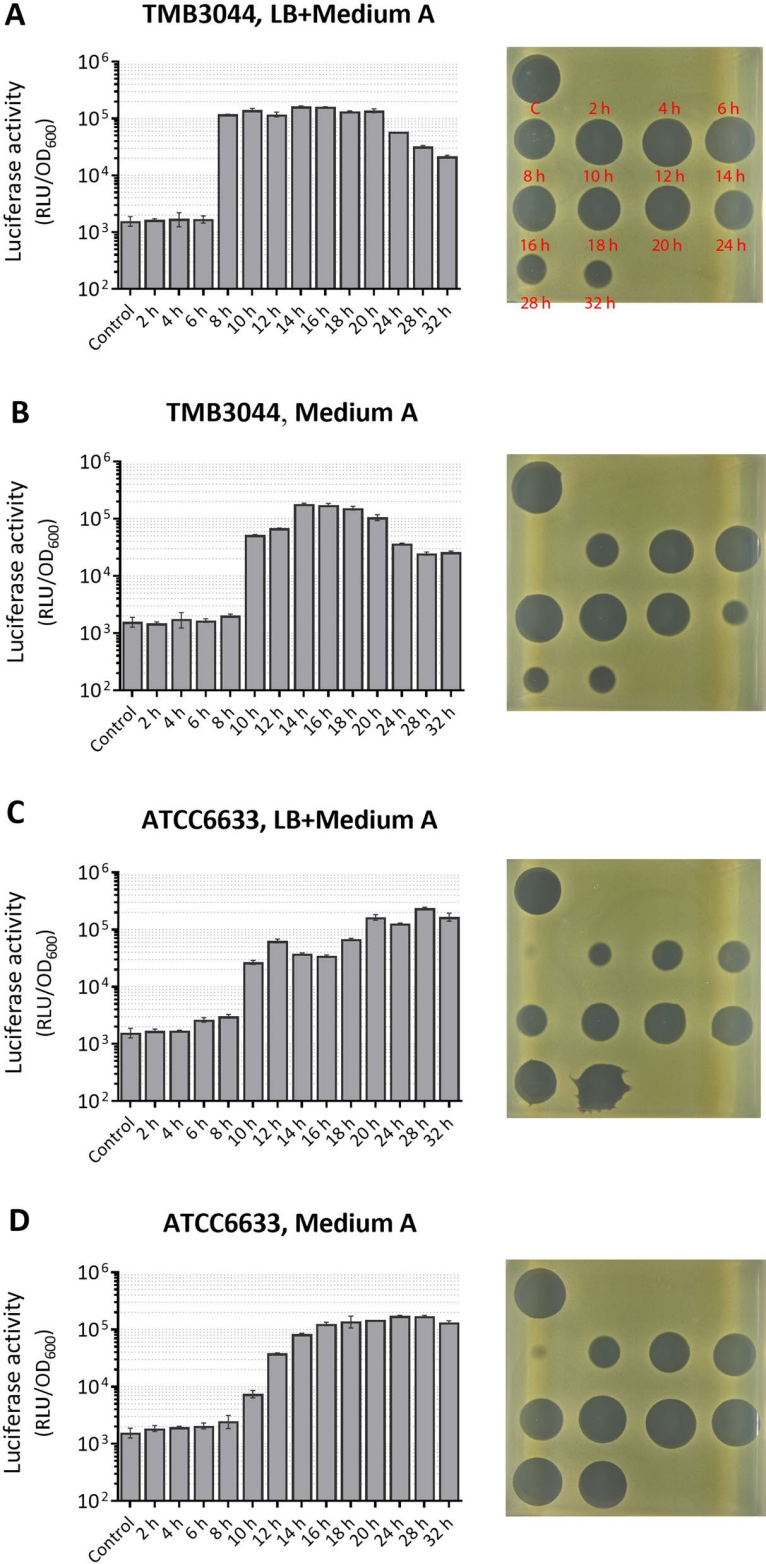
Quantification and antimicrobial activity assay of subtilin production. Strains TMB3044 (W168 *spa* Δ*abrB*) and *B. subtilis* ATCC6633 were cultured in mixed media (50% LB + 50% Medium A) or in Medium A. The liquid culture was collected at defined time points as indicated on the x-axis and filtered to obtain cell-free subtilin supernatant. The subtilin supernatants were tested for subtilin production based on the induction of the reporter strain TMB1617 (W168 P_*liaI*_-*lux*) (left panels). Values are reported as RLU/OD at peak induction point of the reporter as detailed in Additional file 6: Fig. S5. The control was treated with the same amount of sterile water. The antimicrobial activity of the respective subtilin supernatant was tested against the Gram-positive species *M. luteus* (the yellowish soft agar layer), and indicated by the inhibition halo (right panels). The control (labeled as capital letter C) on the left top corner of each plate represents the antimicrobial activity of subtilin supernatant harvested from *B. subtilis* ATCC6633 culture at the 20th hour of growth. Subtilin supernatant under each condition was collected three times and all tested for their antimicrobial activity. The results shown here are representatives for the triplicates of measurement. The error bars in the bar charts represent the standard deviation of triplicates test

Following the preliminary evaluation of subtilin production on solid media, next the subtilin production in liquid was quantified. Here, TMB3044 (W168 *spa* Δ*abrB*) was chosen as the most potent *B. subtilis* W168 subtilin producer, and *B. subtilis* ATCC6633 strain was used as positive control. Three different growth media: (I) 100% LB, (II) 100% Medium A and (III) mixed medium (50% LB + 50% Medium A) were tested to find the best condition for subtilin production in *B. subtilis* W168. Samples of the growing TMB3044 and *B. subtilis* ATCC6633 strains were collected at defined time points and filtered to obtain cell-free subtilin supernatants, which were then evaluated for their ability to induce the reporter strain TMB1617 (P_*liaI*_-*lux*) in microplate reader assays. The activity at the strongest point of induction, as shown in the luminescence curve in Additional file 6: Fig. S5, for each supernatant was depicted in Fig. 6 (left panel) to represent the bioactive subtilin production over the growth.

As shown in Fig. 6 (left panel), TMB3044 was able to produce an amount of bioactive subtilin in both mixed medium and Medium A that was similar to the subtilin amount of *B. subtilis* ATCC6633 growing in Medium A (positive control). Mixed medium was deemed most suitable for TMB3044 given that subtilin was produced earlier and the maximum production sustained longer than in Medium A alone. No detectable subtilin production was obtained in LB culture (Additional file 7: Fig. S6) indicating that some ingredients contained in Medium A were crucial for subtilin biosynthesis. Moreover, the dynamics of subtilin biosynthesis in TMB3044 cultivated in mixed medium (Fig. 6A, left panel) and *B. subtilis* ATCC6633 in Medium A (Fig. 6D, left panel) was consistent with that observed from the “spot-on-lawn” assay, i.e., subtilin was produced earlier by TMB3044 than by *B. subtilis* ATCC6633 and the former reached the maximum of subtilin production more rapidly.

In addition, supernatant samples were tested for their antimicrobial activity against a range of Gram-positive bacteria, including *Micrococcus luteus*, *Staphylococcus aureus* and wild type *B. subtilis* W168, and Gram-negative bacteria including *Escherichia coli* and *Pseudomonas aeruginosa*. To do so, the bacterium to be tested was mixed with soft agar and spread on LB plate as “lawn”. Subtilin supernatants were spotted directly on top. The growth inhibition zones indicate the antimicrobial activity of the corresponding subtilin supernatant. The supernatant samples collected from *B. subtilis* ATCC6633 at the 20th hour of growth in Medium A were used as positive controls. Figure 6 (right panels) shows the results of the growth inhibition assays using *M. luteus* as the indicator strain. Intriguingly, the antimicrobial activity of subtilin supernatant was shown in good correlation with their corresponding ability to induce the P_*liaI*_-reporter strain. Importantly, the supernatants produced by TMB3044 that triggered the same degree of P_*liaI*_ induction as *B. subtilis* ATCC6633 samples also triggered the same degree of antibacterial activity as *B. subtilis* ATCC6633. Furthermore, the antimicrobial activity against other Gram-positive bacteria was also observed, yet to a lesser degree (Additional file 8: Fig. S7). However, not surprisingly, subtilin production was not active against Gram-negative bacteria (data not shown), due to predominantly the outer membrane barrier which bars subtilin from reaching its target lipid II [13, 42].

### “Online” monitoring the dynamic of subtilin production

**Fig. 7:**
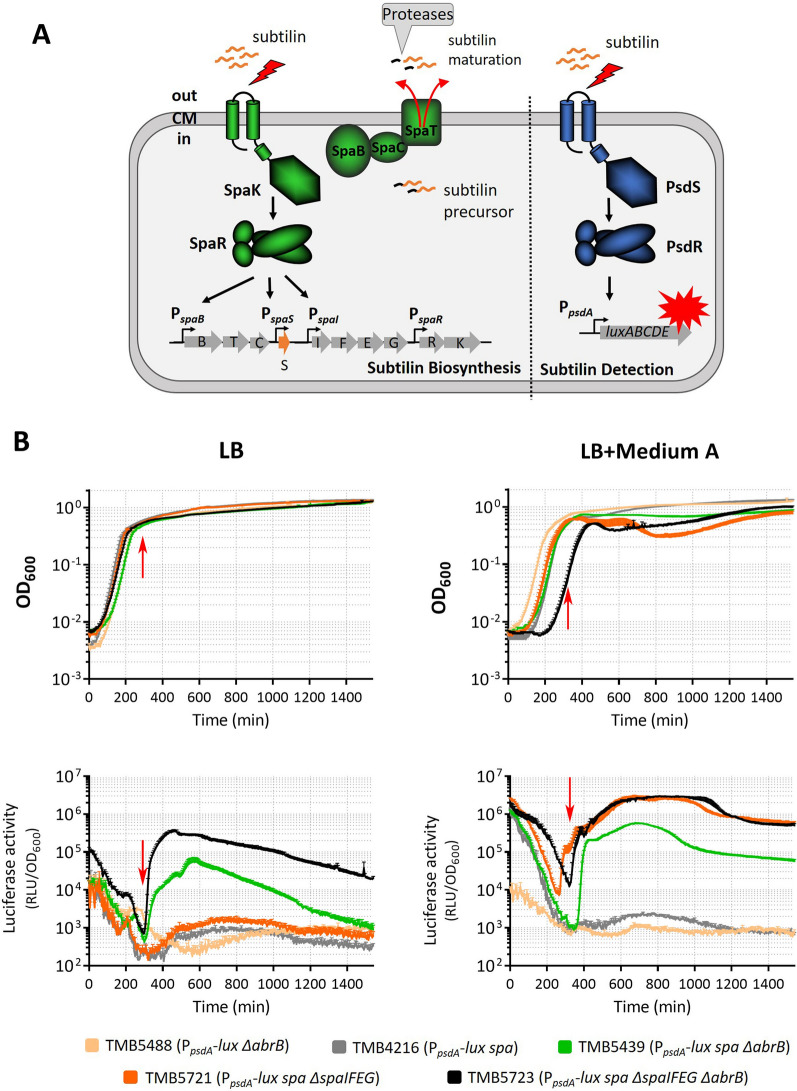
“Online” monitoring the dynamics of subtilin biosynthesis in *B. subtilis* W168. **A** The “online” reporter strain combines the subtilin-producing (*spa*-locus) and -reporting systems (P_*psdA*_-*lux*) in one strain. Subtilin precursor (black and orange wavy line) is encoded by *spaS* and then modified and exported by SpaBTC. The N-terminal leader peptide (black wavy line) is cleaved off by proteases outside of the cell, resulting in bioactive subtilin. The TCS SpaRK is activated by subtilin and then induces subtilin gene expression. Meanwhile, the TCS PsdRS is also activated by active subtilin, inducing P_*psdA*_ and expression of luciferase. Thus, the dynamics of subtilin production can be real-time monitored in the producer itself. **B** “Online” record of subtilin biosynthesis. “Online” reporters were examined in LB and in mixed medium (50% LB + 50% Medium A). The genetic background and the corresponding color code of the strains are given at the bottom of the figure. The red arrows marked in the luminescence graphs indicate the point where the induction starts in TMB5723 strain, while the corresponding arrow in the growth curve indicates the growth status at the same time point. Data are shown as the mean of at least three replicates, with the error bars showing the standard deviations

Although manually collecting cell culture along the growth is generally practical to examine the time course of subtilin biosynthesis, no doubt it is labor-demanding and time-consuming. Therefore, the so-called “online” reporters that combine the subtilin-producing system (*spa*-locus) and subtilin-reporting system (P_*psdA*_-*lux*/P_*liaI*_-*lux*) within one strain were developed to improve the efficiency of monitoring the dynamics of subtilin production. With this, the subtilin molecule secreted outside of the cell is sensed by the reporter system immediately, fulfilling real-time production detection. In this setting, the P_*psdA*_ promoter, which can be induced by AMPs such as nisin and subtilin [43, 44], was chosen in addition to P_*liaI*_ for the construction of a subtilin-reporting system. Similar to P_*liaI*_, the activation of P_*psdA*_ relies on TCS-mediated signal transduction, which in this case is the PsdRS TCS (as depicted in Fig. 7A). The “online” reporter strains were evaluated in three different growth conditions, 100% LB, 100% Medium A and mixed medium (50% LB + 50% Medium A), using a microplate reader (please refer to Material and Methods for details of the assay). The results from P_*psdA*_-based “online” reporters were presented here because they produced a better response behavior to subtilin than the P_*liaI*_-based reporters (Additional file 9: Fig. S8).

As shown in Fig. 7B (bottom panels), TMB5439 (*B. subtilis* W168 P_*psdA*_-*lux spa* Δ*abrB*, green curves) displayed an induction signal at the transition point to stationary phase in both LB and mixed medium conditions, indicating detectable subtilin production at that point. Notably, the induction increased rapidly to the maximum level, especially in mixed medium (~ 233-fold change within 80 min), which could be a result of the quorum-sensing mechanism of subtilin biosynthesis mediated by TCS SpaRK, leading to a positive feedback on subtilin production [45]. In contrast, TMB4261 (W168 P_*psdA*_-*lux spa*) that contains native AbrB did not show induction in any cases, showing again that the presence of AbrB strongly inhibited subtilin biosynthesis. Likewise, no activity of P_*psdA*_ was elicited in TMB5488 (W168 P_*psdA*_-*lux* Δ*abrB*), i.e., a strain lacking the *spa* locus, which ruled out that the induction of P_*psdA*_ activity in strain TMB5439 was caused by *abrB* deletion.

Induction of subtilin production includes the production of the immunity system (SpaIFEG), whose role it is to diminish cell-associated subtilin [18]. It was therefore conceivable that the removal of subtilin by SpaIFEG interfered with detection by the PsdRS-based reporter system. In a next step, we therefore tried to enhance the sensitivity of the “online” reporter by inactivating the immunity genes *spaIFEG*. As indicated in black lines in Fig. 7B, the deletion of *spaIFEG* from TMB5739, resulting in TMB5423 strain (*B. subtilis* W168 P_*psdA*_-*lux spa* Δ*abrB* Δ*spaIFEG*), increased the induction level compared to that in TMB5739. The induction also appeared earlier around the mid-exponential phase, indicating that subtilin was already present in the culture at that time point, but had previously been masked by the activity of SpaIFEG. Indeed, deficiency of the immunity system in TMB4261 could convert this strain (TMB5721, P_*psdA*_-*lux spa* Δ*spaIFEG*, orange curves) into a functional reporter strain in mixed medium despite the presence of AbrB, and the induction was strikingly comparable with that in TMB5723 (black curve). The data suggest that TMB4216 indeed produces subtilin, however the activity of the immunity system SpaIFEG made the small amount of subtilin inaccessible to the sensor of the reporter system, thus failing to produce a detectable signal. The functionality of TMB5721 in mixed medium but not in LB, which was also shown from P_*liaI*_-based “online” reporters (Additional file 9: Fig. S8), highlights again the crucial role of Medium A for subtilin biosynthesis. In summary, the concept of incorporating subtilin-producing and -reporting systems in one strain was successfully substantiated by the development of efficient “online” reporters to monitor the dynamics of subtilin biosynthesis.

## Discussion

**Fig. 8:**
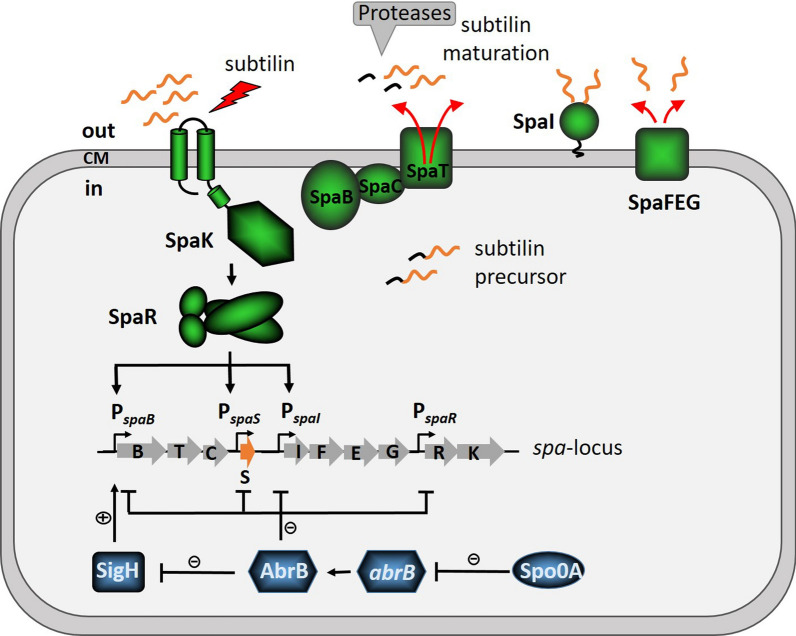
Graphic abstract of the regulation of subtilin biosynthesis in *B. subtilis* W168. Structural gene *spaS* encodes subtilin pre-peptide, which is subjected to post-translational modification by SpaBC and export by SpaT. On the trans side of the cell membrane the leader peptide of subtilin precursor is cleaved off by unspecific proteases, resulting in bioactive subtilin molecule. Subtilin is constitutively expressed at low level, and strongly repressed by transition state regulator AbrB in a SigH-independent manner (except for *spaBTC*). During growth, the cell density is increasing and accompanied by an increased subtilin concentration, which results from the loss of the repression of *spa* genes by AbrB which is repressed by Spo0A as the cell enters transition phase. When a threshold concentration is reached, subtilin acting as a peptide pheromone activates the TCS SpaRK, which in turn induces subtilin gene expression for both subtilin biosynthesis (*spaS* and *spaBTC*) and self-immunity (*spaIFEG*), fulfilling the autoregulatory circuit of subtilin biosynthesis

In the present study, the Gram-positive model organism *B. subtilis* W168 was successfully modified as subtilin producer by integrating the subtilin gene cluster (*spa*-locus) into the genome. The transition state regulator AbrB acted as a strong repressor of subtilin biosynthesis in *B. subtilis* W168. A *B. subtilis* W168 strain carrying the *spa*-locus and *abrB* mutation produced comparable yields of bioactive subtilin as the native producer *B. subtilis* ATCC6633. However, unlike the AbrB in *B. subtilis* ATCC6633, which represses subtilin biosynthesis via SigH, which in turn positively regulates *spaRK* [19], AbrB in *B. subtilis*W168 regulates the majority of *spa* genes in a SigH-independent manner, showing particularly strong control over *spaS*, encoding the subtilin prepeptide. Expression of *spaBTC*, which encode proteins involved in post-translational modification and export of the prepeptide, is significantly controlled by AbrB without the involvement of SigH, while additionally it is also positively regulated by SigH with AbrB acting upstream as a repressor of *sigH*, which turned out to be the only role of SigH in subtilin biosynthesis in *B. subtilis* W168 (Fig. 8).

The results showed that subtilin genes are basally expressed in a growth phase-dependent manner in *B. subtilis* W168 even when subtilin is absent and cannot trigger the auto-inducing loop. Notably, the four transcriptional units contained in the *spa*-locus are expressed at different basal levels in the order, *spaIFEG* < *spaBTC* < *spaS* < *spaRK*. This observation is consistent with the behavior of P_*spaI*_, P_*spaB*_ and P_*spaS*_ reported in *B. subtilis* ATCC6633 [19, 46], while the dynamics of P_*spaR*_ was for the first time characterized here. As a regulatory system controlling the other three transcriptional units, the high basal expression of SpaRK may enable cells to rapidly respond to the accumulated subtilin in the extracellular space, which most likely indicates high cell densities and thus upcoming adverse living conditions in which subtilin production may provide a competitive advantage for survival. The higher amount of subtilin precursor (encoded by *spaS*) than the machinery (encoded by *spaBTC*) for its post-transcriptional modification and export represents a cost-efficient strategy ensuring the amount of substrate exceeds the amount of enzyme. The immunity system-encoding *spaIFEG* shows very low basal expression, which again may be an energy-saving strategy, as immunity is not required unless subtilin concentrations in the environment increase, at which point the auto-induction loop via SpaRK will ensure sufficient immunity gene expression to provide protection.

Subtilin biosynthesis, i.e., the transcription of *spaIFEG*, *spaBTC* and *spaS* is additionally regulated by the TCS SpaRK, which strictly relies on subtilin peptide as inducer to fulfill the autoregulatory circuit. Furthermore, the three corresponding promoters exhibited distinct responsiveness to the activated response regulator SpaR with P_*spaI*_, P_*spaB*_ and P_*spaS*_ strikingly induced by ~ 800-fold, ~ 312-fold and ~ 284-fold by SpaR, respectively. This depicts that the cell, once the auto-induction circuit (via SpaRK) is triggered, can quickly adjust the rest of the Spa system. It massively ramps up immunity to ensure protection, and also increases production of the modification machinery to keep up with modifying the concurrently increased level of pre-peptide.

Intriguingly, the remarkable induction behavior of P_*spaI*_ and its very low basal expression may provide an alternative and more robust setup for the SURE (Subtilin-Regulated Expression) system, which was originally developed based on P_*spaS*_ and SpaRK controlled by its native promoter P_*spaR*_ for protein expression in *B. subtilis* W168 [44, 47]. Furthermore, it should be noted that overproduction of SpaR in *B. subtilis* W168 alone was not sufficient to activate subtilin gene expression. This is in contrast to a previous study in which it was shown that overproduction of SpaR in *Bacillus subtilis* MO1099 (also a derivative of *B. subtilis* 168) could induce subtilin gene expression [19]. Furthermore, overexpression of a TCS response regulator alone to fulfill its functionality seems to be an abundant phenomenon [33–37]. The reason for the infeasibility of SpaR in *B. subtilis* W168 here is unclear, except for the observed difference regarding the overexpression methods. In this study *spaR* was encoded in the genome of *B. subtilis* W168, while in other cases *spaR* was overexpressed from high-copy number plasmid.

Following establishment of the individual components governing heterologous subtilin production, an “online” reporter tool that combines the producing system (*spa*-locus) and reporter system (P_*psdA*_/P_*liaI*_-*lux*) in one strain was developed, which can efficiently and easily monitor the dynamic of subtilin biosynthesis. Importantly, the concept employed here may trigger further strategies for tracking the biosynthesis of many AMPs or antibiotics, if the corresponding reporting systems have been characterized.

In summary, this study reported for the first time the successful heterologous expression of the lantibiotic subtilin in the model organism *B. subtilis* W168 via integrating the complete *spa*-locus into the genome. The regulatory network of subtilin biosynthesis in this host was systematically characterized. The establishment of *B. subtilis* W168 as a platform for subtilin expression will facilitate future genetic (engineering) research on subtilin, as well as pave the way for the industrial consideration of subtilin production. Moreover, the concept employed here will likely be applicable to the study of other lantibiotics and ribosomally synthesized AMPs, especially for those the genetic manipulation in their native producers is still a bottleneck to fulfill in-depth research.

## Materials and methods

### Bacterial strains and growth conditions

Bacterial strains used in this study were routinely grown in Lysogeny Broth (LB) (1% (w/v) tryptone, 0.5% (w/v) yeast extract and 1% (w/v) NaCl) at 37 °C or 30 °C for *M. luteus* with agitation. The subtilin-producing strain *B. subtilis* ATCC6633 was cultivated in high sucrose Medium A [48]. It contains (per liter) 100 g of sucrose, 11.7 g of citric acid, 4 g of Na_2_SO_4_, 4.2 g of (NH_4_)_2_HPO_4_, 5 g of yeast extract, 100 mL of a salt mixture (7.62 g of KCl, 4.18 g of MgCl_2_ × 6H_2_O, 0.543 g of MnCl_2_ × 4H_2_O, 0.49 g of FeCl_3_ × 6H_2_O, and 0.208 g of ZnCl_2_ in 1 L of H_2_O), and sufficient NH_4_OH to bring the pH to 6.8–6.9 per liter. All solid media additionally contained 1.5% (w/v) agar or 0.75% (w/v) for soft agar. MNGE medium was used for *B. subtilis* transformation [49]. Selective media for *B. subtilis* contained kanamycin (10 µg mL^−1^), chloramphenicol (5 µg mL^−1^), spectinomycin (100 µg mL^−1^), tetracycline (12.5 µg mL^−1^) or erythromycin (1 µg mL^−1^) combined with lincomycin (25 µg mL^−1^) for MLS, accordingly. Selective media for *E. coli* contained kanamycin (50 µg mL^−1^), chloramphenicol (35 µg mL^−1^) or ampicillin (100 µg mL^−1^). All strains used in this study are listed in Additional file 1: Table S1.

### DNA manipulation and plasmid construction

General cloning procedure, such as PCR, restriction enzyme digestion and ligation, was performed with enzymes and buffers from New England Biolabs® (NEB, Ipswich, MA, USA) according to the respective instructions. Q5® High-Fidelity DNA polymerase was used for PCRs in case the resulting fragment was further used. Otherwise OneTaq® was the polymerase of choice. PCR-purification was performed using the Hi Yield® PCR Gel Extraction/PCR Clean-up Kit (Süd-Laborbedarf GmbH (SLG), Gauting, Germany). Plasmid preparation was performed using the Hi Yield® Plasmid Mini-kit except for plasmids larger than 10 kb which required the method of alkaline lysis extraction. The *E. coli* competent cells were chemically prepared and transformed using the standard heat-shock method [50]. All the resulting constructs were verified by sequencing. *B. subtilis* transformation was performed as described previously [49]. Successful integration of plasmids into the *B. subtilis* genome was confirmed via colony PCR or on starch plates in the case of *amyE* locus integration events.

To create the integrative plasmid pBS1C-*spaBTCSIFEGRK*, the whole *spa*-locus *spaBTCSIFEGRK* (GenBank: U09819.1) was first amplified from the genomic DNA of *B. subtilis* ATCC6633 strain using primers TM4030 and TM3973. The blunt-ended PCR product of *spa*-locus was then purified and cloned into pCR™-Blunt II-TOPO® vector according to the instruction of Zero Blunt® TOPO® PCR Cloning Kit (Life Technologies). Afterwards, the resulting plasmid pTOPO-*spaBTCSIFEGRK* was cut using type IIS endonuclease *Aar*I to obtain *spa*-locus fragment with *Eco*RI-compatible overhang at the one end and *Spe*I-compatible overhang at the other end, which was then ligated into vector pBS1C cut with *Eco*RI and *Spe*I to generate pBS1C-*spaBTCSIFEGRK*. The pBS1C is one of the empty vectors in the *B. subtilis* BioBrick Box and allows the fragment to be integrated into the *amyE* locus of *B. subtilis* genome [51].

To generate the set of *spa* promoter-*lux* fusions, the *spa* promoters were amplified from the genomic DNA of *B. subtilis* ATCC6633 using respective primer pairs and cloned into pBS3C*lux*, a reporter vector in the *B. subtilis* BioBrick Box [51]. The xylose-inducible promoter P_*xylA*_ was fused to *spaRK* (*spaR*) in the pBS2E vector which allows the integration of the fragment into the *lacA* locus of *B. subtilis* genome [51]. The vectors and the details of plasmid construction are described in Additional file 1: Table S2. Oligonucleotides are given in Additional file 1: Table S3.

### Collection of subtilin-containing supernatant

To prepare subtilin-containing supernatant which was used as inducer across the experiments, 25 mL Medium A was inoculated with a single colony of *B. subtilis* ATCC6633 and grown at 37 °C with agitation. The culture was collected after 24 h of cultivation and centrifuged at 10,000 rcf for 15 min. The supernatant was then sterilized by filtration using 0.45 µm membrane filter (Sarstedt, Germany) and stored at − 20 °C until use.

The heterologous subtilin production in *B. subtilis* W168 was evaluated in three different kinds of media, 100% LB, 100% Medium A and 50% LB + 50% Medium A. The overnight culture of tested strains was diluted 1:1000 into 25 mL of the respective medium. During the cultivation at 37 °C with agitation, 1 mL of the culture was collected every 2 h up to 20 h and subsequently in longer intervals every four hours until 32 h. All the culture samples were filtered using 0.45 µm membrane filter and stored at − 20 °C until use.

### Luciferase assay

The luciferase activity of *B. subtilis* reporter strains carrying *luxABCDE* operon was assayed using a Synergy™ NEO multi-mode microplate reader from BioTek® (Winooski, VT, USA). The reader was controlled by the software Gen5™ (Version 2.06). Luminescence assays were carried out as followed: 10 mL LB medium (w/o antibiotics) were inoculated 1:1000 from overnight cultures (grown with respective antibiotics) and grown to OD_600_ of 0.2–0.3. Then, day cultures were diluted to an OD_600_ of 0.01 and 200 µL were transferred into the wells of a 96-well plate (black wall, clear bottom; Greiner Bio-One, Frickenhausen, Germany). The program was set up for incubation of the plate at 37 °C with agitation (intensity: medium) and the OD_600_ as well as the luminescence was recorded every 5 min for at least 18 h. Luciferase activity (RLU/OD_600_) was defined as the raw luminescence output (relative luminescence units, RLU) normalized to OD_600_ corrected by medium blank at each time point.

If needed, after one hour of incubation, the cells were induced with up to 5 µL of inducer with subtilin supernatant (0.75% (v/v) final concentration) and/or xylose (0.5% (w/v) final concentration). The control samples were treated with the same amount of sterile water. For the “online” reporters, overnight cultures were directly diluted to OD_600_ of 0.01 for luminescence assay without day cultures.

### Spot-on-lawn assay

For this assay, standard LB agar plates were covered with 3.5 mL LB soft agar mixed with 120 µL overnight culture of the reporter strain (as “lawn”) and dried for around 20 min under sterile condition. Afterwards, 6 µL of the overnight culture of the strains (as “spot”) to be tested were spotted on the top of the “lawn” and incubated at 37 °C. Luminescence was evaluated every two hours up to 12 h and subsequently in longer time intervals using a chemiluminescence imaging system FUSION™ (peqlab, VWR International GmbH, Erlangen, Germany). The exposure time was set to 10 min, the aperture was fixed to 0.95 and the full resolution mode was used.

### Growth inhibition experiments

To test the antimicrobial activity of subtilin-containing supernatant, the Gram-positive bacteria, *B. subtilis* W168, *M. luteus* and *S. aureus*, and Gram-negative bacteria *E. coli* and *P. aeruginosa* were the choices of indicator strains. Standard LB agar plates (square, 10 × 10 cm) were overlaid with 6 mL LB soft agar mixed with 200 µL of the overnight culture of the indicator strain, respectively. Afterwards, 6 µL of subtilin supernatants, harvested from potential subtilin-producing *B. subtilis* W168 variants and *B. subtilis* ATCC6633 strain growing in different media, were spotted on the surface of the indicator strain layer. Subsequently, the plates were incubated at 37 °C except for the ones with *M. luteus* which requires 30 °C. The inhibition zone shown indicates the antimicrobial activity of subtilin supernatant.

## Supplementary Information

**Additional file 1: Table S1**. Bacterial strains used in this study. **Table S2**. Plasmids used in this study. **Table S3**. Oligonucleotides used in this study

**Additional file 2: Fig. S1**. Activity of *spa* promoters in absence and presence of *spa*-locus in *B. subtilis* W168. A represents the dynamics of *spa* promoter activity in the absence and presence of *spa*-locus in *B. subtilis* W168 along growth. B represents the response of *spa* promoters to the addition of extra subtilin supernatant in both conditions, showing the existing of *spa*-locus in the strain which caused the SpaRK-mediated autoregulation of subtilin biosynthesis. The color code of the strains is shown on the right side of the respective figure. Luciferase activity was defined as relative luminescence units (RLU) per OD_600_ (RLU/OD_600_). The mean values and standard deviations (error bars) of at least replicate measurements are shown.

**Additional file 3: Fig. S2**. Activity of *spa* promoters in wild type *B. subtilis* W168 and single mutants. A, B, C and D represent the dynamics of *spa* promoter activities in wild type *B. subtilis* W168, Δ*abrB*, Δ*sigH*, and Δ*spo0A* mutants, respectively, in the absence of subtilin. Each of them contains the growth curves on the top, luminescence curves in the middle and the bar graph (as Fig. 2) at the bottom representing the peak luciferase activity shown in each strain. (The following description also applies to Fig. S3, Fig. S4 and Fig. S5) Luciferase activity was defined as relative luminescence units (RLU) per OD_600_ (RLU/OD_600_). The color code of each strain in each panel is depicted at the right side of the growth curves, respectively. The graphs show mean values and standard deviations (error bars) of at least triplicate measurements.

**Additional file 4: Fig. S3**. Activity of *spa* promoters in *B. subtilis* W168 double mutants. A and B represent the dynamics of *spa* promoter activities in W168 Δ*abrB* Δ*sigH* and W168 Δ*abrB* Δ*spo0A* double mutants, respectively, in the absence of subtilin. Each of them contains the growth curves on the top, luminescence curves in the middle and the bar graph (as Fig. 3) at the bottom representing the peak luciferase activity in each strain.

**Additional file 5: Fig. S4**. Regulation of the two-component system (TCS) SpaRK on subtilin biosynthesis. A and B represent the dynamics of spa promoter activities in *B. subtilis* W168 P_*xylA*_-*spaRK* and *B. subtilis* W168 P_*xylA*_-*spaR* under different treatment conditions (untreated, treated with xylose and treated with both xylose and subtilin), respectively. P_*xylA*_ indicates a xylose-inducible promoter. Each of them contains the growth curves on the top, luminescence curves in the middle and the bar graph (as Fig. 4) at the bottom representing the peak luciferase activity in each strain.

**Additional file 6: Fig. S5**. Quantification of subtilin production. The subtilin supernatants collected from TMB3044 (W168 *spa* Δ*abrB*) and subtilin native producer *B. subtilis* ATCC6633 at defined time points of growth as indicated at the right side of the growth curves were tested using reporter strain TMB1617 (P_*liaI*_-*lux*). Mixed medium (50% LB + 50% Medium A) and Medium A were used for the cultivation, respectively, for both strains. The subtilin supernatant was added to the cell culture after 1 hour of growth in a microplate reader. Each of the A, B, C and D contains the growth curves on the top, luminescence curves in the middle and the bar graph (as Fig. 6) at the bottom representing the peak luciferase activity triggered by corresponding subtilin supernatant.

**Additional file 7: Fig. S6**. Evaluation of subtilin production of TMB3044 cultured in LB medium. The subtilin supernatants collected at defined time points from TMB3044 (W168 *spa* Δ*abrB*) cultured in LB medium were tested using reporter strain TMB1617 (P_*liaI*_-*lux*). Control (-) indicates instead of subtilin supernatant the reporter strain was treated with the same volume of sterile water. Control (+) as the positive control was treated with 0.75% subtilin supernatant collected from *B. subtilis* ATCC6633 at the 20th hour of growth in Medium A. The upper panel shows the growth curves of the reporter stain under different treatments, and the bottom panel represents the luminescence curves under the corresponding treatments. The graphs show mean values and standard deviations (error bars) of three biological replicates

**Additional file 8: Fig. S7**. Antimicrobial activity of subtilin against G^+^ bacteria *S. aureus* and *B. subtilis* W168. The same subtilin supernatant used in Fig. 6 were applied here, including the subtilin supernatant collected from TMB3044 (W168 *spa* Δ*abrB*) and *B. subtilis* ATCC6633 strain growing in mixed medium (50% LB + 50% Medium A), and 100% Medium A at defined time points as labeled on the top left plate in red. The control (indicated as C) was the subtilin supernatant collected from *B. subtilis* ATCC6633 at the 20th hour of growth in Medium A.

**Additional file 9: Fig. S8**. “Online” monitoring the subtilin biosynthesis in *B. subtilis* W168 (P_*liaI*_-*lux* as the reporting system). The rationale of the response of P_*liaI*_ to subtilin is given in Fig. 7A. “Online” reporters were examined in LB and mixed medium (50% LB + 50% Medium A), respectively. The genetic background and color-code of the strains are given at the bottom of the figure. The red arrows marked in luminescence graphs indicate the point where the induction starts in TMB5722 strain, while the corresponding arrow in the growth curve indicates the growth situation at the same time point. The mean of at least three replicates is shown with the error bars indicating the standard deviations.

## Data Availability

All the data generated and analyzed during this study are included in the manuscript and its Additional files.
