# Supplementary material for: Regulation of heterologous subtilin production in Bacillus subtilis W168
Source: Microb Cell Fact. 2022 Apr 7;21:57. doi: 10.1186/s12934-022-01782-9 (PMC8991943; doi:10.1186/s12934-022-01782-9)
Supplement: Supplementary file 1 — Additional file 1: Table S1. Bacterial strains used in this study. Table S2. Plasmids used in this study. Table S3. Oligonucleotides used in this study [file 12934_2022_1782_MOESM1_ESM.docx]

**Additional file 2:**

Table S1 Bacterial strains used in this study.

| **Stain** | **Description^a^** | | **Source/Reference^b^** |
| --- | --- | --- | --- |
| *Bacillus subtilis* |  | |  |
| W168 | Wild type, *trpC2* | | Laboratory stock [1] |
| ATCC6633 | Subtilin producer | | Laboratory stock |
| 1S143 | W168 *spo0A*::*kan*, *kan*^r^ | | BGSC |
| TMB082 | W186 *abrB*::*kan*, *kan*^r^ | | Laboratory stock |
| TMB205 | W168 *spo0A*::*tet*, *tet*^r^ | | Laboratory stock |
| TMB1617 | W168 *sacA*::P*_liaI_*-*lux*, *cm*^r^ | | Laboratory stock |
| TMB1619 | W168 *sacA*::P*_bceA_*-*lux*, *cm*^r^ | | Laboratory stock |
| TMB2009 | W168 *sacA*::P*_psdA_*-*lux*, *cm*^r^ | | Laboratory stock |
| TMB2841 | W168 *sacA*::P*_empty_*-*lux*, *cm*^r^ | | Laboratory stock |
| TMB3039 | W168 *amyE*::spaBTCSIFEGRK, *cm*^r^ | | This study |
| TMB3044 | W168 *abrB*::*kan* *amyE*::spaBTCSIFEGRK, *kan*^r^, *cm*^r^ | | This study |
| TMB3045 | W168 *sigH*::*tet*, *tet*^r^ | | This study |
| TMB3052 | W168 *spo0A*::*kan* *amyE*::spaBTCSIFEGRK, *kan*^r^, *cm*^r^ | | This study |
| TMB3057 | W168 *sigH*::*tet* *amyE*::spaBTCSIFEGRK, *tet*^r^, *cm*^r^ | | This study |
| TMB3627 | W168 *sacA*::P*_spaR_*-*lux*, *kan*^r^ | | This study |
| TMB3652 | W168 *sacA*::P*_spaR_*-*lux* *lacA*:: P*_xylA_*-*spaR*, *kan*^r^, *mls*^r^ | | This study |
| TMB3663 | W168 *sacA*::P*_spaS_*-*lux*, kan^r^ | | This study |
| TMB3665 | W168 *sacA*::P*_spaS_*-*lux lacA*:: P*_xylA_*-*spaR*, *kan*^r^, *mls*^r^ | | This study |
| TMB3672 | W168 *sacA*::P*_spaS_*-*lux* *amyE*::spaBTCSIFEGRK, *kan*^r^, *cm*^r^ | | This study |
| TMB3673 | W168 *sacA*::P*_spaI_*-*lux*, *kan*^r^ | | This study |
| TMB3697 | W168 *sacA*::P*_spaS_*-*lux* *sigH*::*tet*, *kan*^r^, *tet*^r^ | | This study |
| TMB3698 | W168 *sacA*::P*_spaI_*-*lux* *sigH*::*tet*, *kan*^r^, *tet*^r^ | | This study |
| TMB3699 | W168 *sacA*::P*_spaS_*-*lux* *amyE*::spaBTCSIFEGRK *sigH*::*tet*, *kan*^r^, *cm*^r^, *tet*^r^ | | This study |
| TMB3702 | W168 *sacA*::P*_spaI_*-*lux* *lacA*:: P*_xylA_*-*spaR*, *kan*^r^, *mls*^r^ | | This study |
| TMB3706 | W168 *sacA*::P*_spaI_*-*lux* *spo0A*::*tet*, *kan*^r^, *tet*^r^ | | This study |
| TMB3707 | W168 *sacA*::P*_spaS_*-*lux* *spo0A*::*tet*, *kan*^r^, *tet*^r^ | | This study |
| TMB3708 | W168 *sacA*::P*_spaI_*-*lux* *amyE*::spaBTCSIFEGRK *spo0A*::*tet*, *kan*^r^, *cm*^r^, *tet*^r^ | | This study |
| TMB3710 | W168 *sacA*::P*_spaB_*-*lux*, *kan*^r^ | | This study |
| TMB3736 | W168 *sacA*::P*_spaI_*-*lux* *amyE*::spaBTCSIFEGRK, *kan*^r^, *cm*^r^ | | This study |
| TMB3741 | W168 *sacA*::P*_spaB_*-*lux* *sigH*::*tet*, *kan*^r^, *tet*^r^ | | This study |
| TMB3742 | W168 *sacA*::P*_spaB_*-*lux* *amyE*::spaBTCSIFEGRK, *kan*^r^, *cm*^r^ | | This study |
| TMB3745 | W186 *abrB*::*spec*, *spec*^r^ | | This study |
| TMB3748 | W168 *sacA*::P*_spaB_*-*lux* *spo0A*::*tet*, *kan*^r^, *tet*^r^ | | This study |
| TMB3751 | W168 *sacA*::P*_spaB_*-*lux* *lacA*:: P*_xylA_*-*spaR*, *kan*^r^, *mls*^r^ | | This study |
| TMB3753 | W168 *sacA*::P*_spaR_*-*lux* *spo0A*::*tet*, *kan*^r^, *tet*^r^ | | This study |
| TMB3772 | W168 *sacA*::P*_spaR_*-*lux* *sigH*::*tet*, *kan*^r^, *tet*^r^ | | This study |
| TMB3773 | W168 *sacA*::P*_spaI_*-*lux* *amyE*::spaBTCSIFEGRK *sigH*::*tet*, *kan*^r^, *cm*^r^, *tet*^r^ | | This study |
| TMB3774 | W168 *sacA*::P*_spaB_*-*lux* *amyE*::spaBTCSIFEGRK *sigH*::*tet*, *kan*^r^, *cm*^r^, *tet*^r^ | | This study |
| TMB3775 | W168 *sacA*::P*_spaS_*-*lux* *abrB*::*spec*, *kan*^r^, *spec*^r^ | | This study |
| TMB3776 | W168 *sacA*::P*_spaI_*-*lux* *abrB*::*spec*, *kan*^r^, *spec*^r^ | | This study |
| TMB3778 | W168 *sacA*::P*_spaB_*-*lux* *abrB*::*spec*, *kan*^r^, *spec*^r^ | | This study |
| TMB3779 | W168 *sacA*::P*_spaR_*-*lux* *abrB*::*spec*, *kan*^r^, *spec*^r^ | | This study |
| TMB3781 | W168 *sacA*::P*_spaS_*-*lux* *amyE*::spaBTCSIFEGRK *abrB*::*spec*, *kan*^r^, *cm*^r^, *spec*^r^ | | This study |
| TMB3782 | W168 *sacA*:: P*_spaB_*-*lux* *amyE*::spaBTCSIFEGRK *abrB*::*spec*, *kan*^r^, *cm*^r^, *spec*^r^ | | This study |
| TMB3783 | W168 *sacA*:: P*_spaI_*-*lux* *amyE*::spaBTCSIFEGRK *abrB*::*spec*, *kan*^r^, *cm*^r^, *spec*^r^ | | This study |
| TMB4128 | W168 *sacA*:: P*_spaR_*-*lux* *amyE*::spaBTCSIFEGRK, *kan^r^*, *cm*^r^ | | This study |
| TMB4197 | W168 *sacA*::P*_spaI_*-*lux* *lacA*::P*_xylA_*-*spaRK*, *kan*^r^, *mls*^r^ | | This study |
| TMB4198 | W168 *sacA*::P*_spaB_*-*lux* *lacA*::P*_xylA_*-*spaRK*, *kan*^r^, *mls*^r^ | | This study |
| TMB4199 | W168 *sacA*::P*_spaS_*-*lux* *lacA*::P*_xylA_*-*spaRK*, *kan*^r^, *mls*^r^ | | This study |
| TMB4200 | W168 *sacA*::P*_spaR_*-*lux* *lacA*::P*_xylA_*-*spaRK*, *kan*^r^, *mls*^r^ | | This study |
| TMB4215 | W168 *sacA*::P*_liaI_*-*lux* *amyE*::spaBTCSIFEGRK, *cm*^r^, *kan*^r^ | | This study |
| TMB4216 | W168 *sacA*::P*_psdA_*-*lux* *amyE*::spaBTCSIFEGRK, *cm*^r^, *kan*^r^ | | This study |
| TMB4225 | W168 *sacA*::P*_spaR_*-*lux* *amyE*::spaBTCSIFEGRK *sigH*::*tet*, *kan*^r^, *cm*^r^, *tet*^r^ | | This study |
| TMB4226 | W168 *sacA*::P*_spaB_*-*lux* *amyE*::spaBTCSIFEGRK *spo0A*::*tet*, *kan*^r^, *cm*^r^, *tet*^r^ | | This study |
| TMB4227 | W168 *sacA*::P*_spaS_*-*lux* *amyE*::spaBTCSIFEGRK *spo0A*::*tet*, *kan*^r^, *cm*^r^, *tet*^r^ | | This study |
| TMB4228 | W168 *sacA*::P*_spaR_*-*lux* *amyE*::spaBTCSIFEGRK *spo0A*::*tet*, *kan*^r^, *cm*^r^, *tet*^r^ | | This study |
| TMB4229 | W168 *sacA*::P*_spaR_*-*lux* *amyE*::spaBTCSIFEGRK *abrB*::*spec*, *kan*^r^, *cm*^r^, *spec*^r^ | | This study |
| TMB4230 | W168 *amyE*::spaBTCSIFEGRK, *kan*^r^ | | This study |
| TMB5438 | W168 *sacA*::P*_liaI_*-*lux* *amyE*::spaBTCSIFEGRK *abrB*::*spec*, *cm*^r^, *kan*^r^, *spec*^r^ | | This study |
| TMB5439 | W168 *sacA*::P*_psdA_*-*lux* *amyE*::spaBTCSIFEGRK *abrB*::s*pec*, *cm*^r^, *kan*^r^, *spec*^r^ | | This study |
| TMB5479 | W168 *sacA*::P*_spaI_*-*lux* *abrB*::*spec sigH*::*tet*, *kan*^r^, *spec*^r^, *tet*^r^ | | This study |
| TMB5480 | W168 *sacA*::P*_spaB_*-*lux* *abrB*::*spec sigH*::*tet*, *kan*^r^, *spec*^r^, *tet*^r^ | | This study |
| TMB5481 | W168 *sacA*::P*_spaS_*-*lux* *abrB*::*spec sigH*::*tet*, *kan*^r^, *spec*^r^, *tet*^r^ | | This study |
| TMB5482 | W168 *sacA*::P*_spaR_*-*lux* *abrB*::*spec sigH*::*tet*, *kan*^r^, *spec*^r^, *tet*^r^ | | This study |
| TMB5483 | W168 *sacA*::P*_spaI_*-*lux* *abrB*::*spec* *spo0A*::*tet*, *kan*^r^, *spec*^r^, *tet*^r^ | | This study |
| TMB5484 | W168 *sacA*::P*_spaB_*-*lux* *abrB*::*spec* *spo0A*::*tet*, *kan*^r^, *spec*^r^, *tet*^r^ | | This study |
| TMB5485 | W168 *sacA*::P*_spaS_*-*lux* *abrB*::*spec* *spo0A*::*tet*, *kan*^r^, *spec*^r^, *tet*^r^ | | This study |
| TMB5486 | W168 *sacA*::P*_spaR_*-*lux* *abrB*::*spec* *spo0A*::*tet*, *kan*^r^, *spec*^r^, *tet*^r^ | | This study |
| TMB5487 | W168 *sacA*::P*_liaI_*-*lux* *abrB*::*spec*, *cm*^r^, *spec*^r^ | | This study |
| TMB5488 | W168 *sacA*::P*_psdA_*-*lux* *abrB*::*spec*, *cm*^r^, *spec*^r^ | | This study |
| TMB5489 | W168 *sacA*::P*_bceA_*-*lux* *abrB*::*spec*, *cm*^r^, *spec*^r^ | | This study |
| TMB5490 | W168 *sacA*::P*_bceA_*-*lux* *amyE*::*spaBTCSIFEGRK* *abrB*::*spec*, *cm*^r^, *kan*^r^, *spec*^r^ | | This study |
| TMB5491 | W168 *sacA*::P*_bceA_*-*lux* *amyE*::*spaBTCSIFEGRK*, *cm*^r^, *kan*^r^ | | This study |
| TMB5720 | W168 *sacA*::P*_liaI_*-*lux* *amyE*::*spaBTCSIFEGRK* *spaIFEG*::*mls*, *kan*^r^, *cm*^r^, *mls*^r^ | | This study |
| TMB5721 | W168 *sacA*::P*_psdA_*-*lux* *amyE*::*spaBTCSIFEGRK* *spaIFEG*::*mls*, *kan*^r^, *cm*^r^, *mls*^r^ | | This study |
| TMB5722 | W168 *sacA*::P*_liaI_*-*lux* *amyE*::*spaBTCSIFEGRK* *abrB*::*spec* *spaIFEG*::*mls*, *kan*^r^, *cm*^r^, *spec*^r^*, mls*^r^ | | This study |
| TMB5723 | W168 *sacA*::P*_psdA_*-*lux* *amyE*::*spaBTCSIFEGRK* *abrB*::*spec* *spaIFEG*::*mls*, *kan*^r^, *cm*^r^, *spec*^r^*, mls*^r^ | | This study |
| ***Escherichia coli*** |  | |  |
| DH5α | *E. coli F– endA1 glnV44 thi-1 recA1 relA1 gyrA96 deoR nupG purB20 φ80dlacZ*Δ*M15* Δ*(lacZYA-argF)U169, hsdR17(rK–mK+), λ–* | | Laboratory stock |
| DH10β | *E. coli F– endA1 deoR+ recA1 galE15 galK16 nupG rpsL* Δ*(lac)X74 φ80lacZ*Δ*M15 araD139* Δ*(ara,leu)7697 mcrA* Δ*(mrr-hsdRMS-mcrBC) StrR λ–* | | Laboratory stock |
| XL1 blue | *E. coli endA1 gyrA96(nalR) thi-1 recA1 relA1 lac glnV44 F'[ ::Tn10 proAB+ lacIq* Δ*(lacZ)M15] hsdR17(rK- mK+)* | | Laboratory stock |
| TME956 | XL1 blue pSB1C3-[RFP], *cm*^r^ | | iGEM Registry |
| TME1087 | XL1 blue pSB1A3-P*_xylA_*, *cm*^r^ | | Laboratory stock |
| TME1905 | DH5α pCKSBC107 (pSB1C3-P*_spaR_*), *cm*^r^ | | This study |
| TME1906 | DH5α pCKSBC104 (pSB1C3-*spaRK*), *cm*^r^ | | This study |
| TME1924 | DH5α TopoCK01 (Topo-*spaBTCSIFEGRK*), *kan*^r^ | | This study |
| TME1925 | DH5α pCK1C01 (pBS1C-*spaBTCSIFEGRK*), *amp*^r^ | | This study |
| TME1927 | DH5α pCK2E02 (pBS2E-P*_xylA_*-*spaRK*), *amp*^r^ | | This study |
| TME2425 | DH10β pVG2EF02 (pBS2E-P*_xylA_*-*spaR*), *amp*^r^ | | This study |
| TME2463 | DH10ß pJE3Klux01 (pBS3K*lux*-P*_spaR_*), *amp*^r^ | | This study |
| TME2478 | DH10ß pJE3Klux02 (pBS3K*lux*-P*_spaI_*), *amp*^r^ | | This study |
| TME2511 | DH10ß pJE3Klux03 (pBS3K*lux*-P*_spaS_*), *amp*^r^ | | This study |
| TME2527 | DH10β pVG3Klux01 (pBS3K*lux*-P*_spaB_*), *amp*^r^ | | This study |
| TME2951 | DH10ß pBS1K-*spaBTCSIFEGRK*, *amp*^r^ | | This study |
| **Other strains** |  | |  |
| *Micrococcus luteus* | | Wild type | Laboratory stock |
| *Staphylococcus aureus* | | Wild type | Laboratory stock |
| *Pseudomonas aeruginosa* | | Wild type | Laboratory stock |

^a^ amp^r^: resistance to ampicillin, cm^r^: resistance to chloramphenicol, kan^r^: resistance to kanamycin, mls^r^: resistance to erythromycin/lincomycin, spec^r^: resistance to spectinomycin, tet^r^: resistance to tetracyclin, *lux*: *luxABCDE*

^b^ BGSC: Bacillus Genetic Stock Center (http://www.bgsc.org), iGEM Registry: iGEM Registry of Standard Biological Parts (http://parts.igem.org).

Table S2 Vectors and plasmids used in this study.

| **Plasmids** | **Description^a^** | **Source/Reference^b^** |
| --- | --- | --- |
| **Vectors** |  |  |
| pCR^TM^ Blunt II-TOPO^®^ | Insertion of blunt-ended PCR products, *lacZα-ccdB*, *kan*^r^ | Life technologies |
| pSB1C3 | Empty vector, ori pMB1(high copy number), cm^r^ | iGEM Registry |
| pBS3K*lux* | *lux*-reporter vector, integration at *sacA*, *amp*^r^, *kan*^r^ | [2] |
| pBS2E | Empty vector, integration at *lacA*, *amp*^r^, *mls*^r^ | [3] |
| pBS1C | Empty vector, integration at *amyE*, *amp*^r^, *cm*^r^ | [3] |
| pBS1K | Empty vector, integration at *amyE*, *amp*^r^, *kan*^r^ | [2] |
| pDG1513 | Source of *tet* cassette for LFH-PCR | [4] |
| pDG1726 | Source of *spec* cassette for LFH-PCR | [4] |
| **Plasmids** |  |  |
| 2161 pBS3K*lux*-P*_spaI_* | P*_spaI_* (TM4035-4040 from *B. subtilis* ATCC6633, E+S) in pBS3K*lux* (E+S), *amp*^r^, *kan*^r^ | This study |
| 2201 pBS3K*lux*-P*_spaB_* | P*_spaB_*(TM4031-4032 from *B. subtilis* ATCC6633, E+P) in pBS3K*lux* (E+P), *amp*^r^, *kan*^r^ | This study |
| 2187 pBS3K*lux*-P*_spaS_* | P*_spaS_* (TM4033-4034 from *B. subtilis* ATCC6633, E+S) in pBS3K*lux* (E+S), *amp*^r^, *kan*^r^ | This study |
| 2148 pBS3K*lux*-P*_spaR_* | P*_spaR_* (pl. 1723, E+S) in pBS3K*lux* (E+S), *amp*^r^, *kan*^r^ | This study |
| 1723 pSB1C3-P*_spaR_* | P*_spaR_* (TM4044-4045 from *B. subtilis* ATCC6633, E+P) in pSB1C3 (E+P), *cm*^r^ | This study |
| 1720 pSB1C3-*spaRK* | spaRK (TM4024-4029 from *B. subtilis* ATCC6633, *Bsa*I) in pSB1C3 (E+S), *cm*^r^ | This study |
| 1076 pSB1A3-P*_xylA_* | xylose-inducible promoter P*_xylA_* donor, *cm^r^* | Laboratory stock |
| 2138 pBS2E-P*_xylA_*-*spaR* | P*_xylA_* (pl. 1076, E+S) and *spaR* (TM4986-4987 from pl. 1720, X+P) in pBS2E (E+P), *cm*^r^, *mls*^r^ | This study |
| 1735 pBS2E-P*_xylA_*-*spaRK* | P*_xylA_* (pl. 1076, E+S) and *spaRK* (pl. 1720, X+P) in pBS2E (E+P), *cm*^r^, *mls*^r^ | This study |
| 1730 pTOPO-*spaBTCSIFEGRK* | *spaBTCSIFEGRK* (TM4030/TM3973 from *B. subtilis* ATCC6633) in pCR^TM^ Blunt II-TOPO^®^, *kan*^r^ | This study |
| 1731 pBS1C- *spaBTCSIFEGRK* | *spaBTCSIFEGRK* (*Aar*I from pTOPO-*spaBTCSIFEGRK*) in pBS1C (E+S), *amp*^r^, *cm*^r^ | This study |
| 2538 pBS1K-*spaBTCSIFEGRK* | *spaBTCSIFEGRK* (*Aar*I from TOPO-*spaBTCSIFEGRK*) in pBS1K (E+S), *amp*^r^, *kan*^r^ | This study |

^a^ amp^r^: resistance to ampicillin, cm^r^: resistance to chloramphenicol, kan^r^: resistance to kanamycin, mls^r^: resistance to erythromycin/lincomycin, *lux*: *luxABCDE*, E: EcoRI, P: PstI, S: SpeI, X: XbaI.

^b^ BGSC: Bacillus Genetic Stock Center (<http://www.bgsc.org>), iGEM Registry: iGEM Registry of Standard Biological Parts (<http://parts.igem.org>), Life Technologies (Waltham, MA, USA)

Table S3 Oligonucleotides used in this study.

| **Primer** | **Description^a^** | **Primer sequence 5’→3’^b^** |
| --- | --- | --- |
| TM0056 | kan-check fwd | CATCCGCAACTGTCCATACTCTG |
| TM0141 | *spec* fwd (LFH-*spec*-cassette) | CAGCGAACCATTTGAGGTGATAGGGACTGGCTCGCTAATAACGTAACGTGACTGGCAAGAG |
| TM0142 | *spec* rev (LFH-*spec*-cassette) | CGATACAAATTCCTCGTAGGCGCTCGGCGTAGCGAGGGCAAGGGTTTATTGTTTTCTAAAATCTG |
| TM0144 | Tc fwd2 (LFH-*tet*-cassette) | CAGCGAACCATTTGAGGTGATAGGGCTTATCAACGTAGTAAGCGTGG |
| TM0145 | Tc rev (LFH-*tet*-cassette) | CGATACAAATTCCTCGTAGGCGCTCGGGAACTCTCTCCCAAAGTTGATCCC |
| TM0281 | *abrB*-up fwd | TATCAACGAGCTGAGTTTCCG |
| TM0282 | *abrB*-up rev | CCTATCACCTCAAATGGTTCGCTGCAACTTTACGTACAATACCAGTAG |
| TM0283 | *abrB*-do fwd | CGAGCGCCTACGAGGAATTTGTATCGCAGCGAAATCCAAAACCAGC |
| TM0284 | *abrB*-do rev | TTCTTTACTTGGTCCCAACCC |
| TM0307 | *spo0A*-up fwd | TATCAGAGATTCTGCTGCTGGC |
| TM0308 | *spo0A*-up rev | CCTATCACCTCAAATGGTTCGCTGAGCGACAGGCATTCCTGTCC |
| TM0309 | *spo0A*-do fwd | CGAGCGCCTACGAGGAATTTGTATCGGTTGCGGATAAGCTGAGG |
| TM0310 | *spo0A*-do rev | GGAAGAACCTGAGACACCG |
| TM0749 | pSWEET-amyE-check rev  (pBS1C check) | AAAGGTCATTGTTGACGCGG |
| TM2262 | pAH328checkfwd  (pBS1C/pBS3K*lux* check) | GAGCGTAGCGAAAAATCC |
| TM2263 | pAH328checkrev  (pBS3K*lux* check) | GAAATGATGCTCCAGTAACC |
| TM2505 | pAH328 *sacA* front check fwd | CTGATTGGCATGGCGATTGC |
| TM2506 | pAH328 sacA front check rev | ACAGCTCCAGATCCTCTACG |
| TM2507 | pAH328 *sacA* back check fwd | GTCGCTACCATTACCAGTTG |
| TM2508 | pAH328 *sacA* back check rev | TCCAAACATTCCGGTGTTATC |
| TM2889 | seqcheck fwd (pSB1C3) | TGCCACCTGACGTCTAAG |
| TM2890 | seqcheck rev (pSB1C3) | ATTACCGCCTTTGAGTGA |
| TM3026 | M13 rev  (seqcheck TOPO-Vector) | CAGGAAACAGCTATGAC |
| TM3027 | M13 fwd (-20)  (seqcheck TOPO-Vector) | GTAAAACGACGGCCAG |
| TM3081 | pSBBs2E seq fwd | GGCAACCGAGCGTTCTG |
| TM3082 | pSBBs2E seq rev | CTGACAGCGTTTCGATCC |
| TM3973 | SpaOperon_AarI_S_rev | CACCACCTGCCGGACTAGTTTATTCGTTTCTTAAAGGTATTTTTACTCG |
| TM4024 | SpaRK_BsaI_ENX_SD_NgoMIV_fwd | GGAGGTCTCGAATTCGCGGCCGCTTCTAGATAAGGAGGAGCCGGCATGGCAAAAATCCTTGCTGTCGATGATG |
| TM4025 | SpaRK_BsaI_PstI11275mut_rev | CACGGTCTCCTGAAGATCTTTTTCTTTAATAGTTCCAGCTG |
| TM4026 | SpaRK_BsaI_PstI11275mut_fwd | CACGGTCTCCTTCAGCTGGTAAAAAAGAAGATTGAAAAAAAACC |
| TM4027 | SpaRK_BsaI_EcoRI11602mut_rev | CACGGTCTCCAACTCCTTAATATGTGTAAATTCGGGAGTG |
| TM4028 | SpaRK_BsaI_EcoRI11602mut_fwd | GGAGGTCTCCAGTTCGATGATGTGATTGACTCGTTGATTG |
| TM4029 | SpaRK_BsaI_A_S_rev | CACGGTCTCACTAGTATTATGGCCATTCGTTTCTTAAAGGTATTTTTACTCGAACCTC |
| TM4030 | SpaOperon_AarI_E_fwd_+PspaB | GGACACCTGCTACGAATTCCGCTCGCGCTTTCTGTCATTATG |
| TM4031 | PspaB_ENX_fwd | TGCAGAATTCGCGGCCGCTTCTAGAGCGCTCGCGCTTTCTGTCATTATG |
| TM4032 | PspaB_SNP_rev | AGCCTGCAGCGGCCGCTACTAGTACATATTTATCCGCTTCTTAATAAAAACAATTC |
| TM4033 | PspaS_ENX_fwd | TGCAGAATTCGCGGCCGCTTCTAGAGCAGAGATGCTTGGTGTTATGGAAG |
| TM4034 | PspaS_SNP_rev | AGCCTGCAGCGGCCGCTACTAGTATCAATACCTAAATAGTAACAGACAAATATCAAG |
| TM4035 | PspaI_BsaI_ENX_fwd | GGAGGTCTCGAATTCGCGGCCGCTTCTAGAGCATAGAGTCGGGACAAGAAAATGAAGTAAAAAACG |
| TM4036 | PspaI_BsaI_XbaI7321mut_rev | CACGGTCTCCCTAAATGGATCAATCTCATTTATTAATTTCCTAAT |
| TM4037 | PspaI_BsaI_XbaI7321mut_fwd | GGAGGTCTCCTTAGAGTAGGAGTGTGACCTGATCG |
| TM4038 | PspaI_BsaI_XbaI7444mut_rev | CACGGTCTCCCTGGAATACTTTTATCAAAATTTAATCAAAAAAACATTTCC |
| TM4039 | PspaI_BsaI_XbaI7444mut_fwd | GGAGGTCTCCCCAGAATGGTCTGCATCCGGAAAAAAGG |
| TM4040 | PspaI_BsaI_S_rev | CACGGTCTCACTAGTAATGAATTTTCCTCAATAGATGTTAAGCAATAATGG |
| TM4044 | PspaR_ENX_fwd | TGCAGAATTCGCGGCCGCTTCTAGAGGTATTACTAGGGAGCACATCTCCG |
| TM4045 | PspaR_SNP_rev | AGCCTGCAGCGGCCGCTACTAGTAATACTATTCAAACGTTTTTCTGCCTTCC |
| TM4085 | pBS2E int. up fwd | TGCTGCAAAAGAATTTTGTGTCCG |
| TM4086 | pBS2E int. up rev | AGGACTCTCTAGCTTGAGGC |
| TM4087 | pBS2E int. do fwd | CTGCAGAGATATCGATTTCAAGC |
| TM4088 | pBS2E int. do rev | CTTTGCTTTTCATGATTTCATCCC |
| TM4175 | *sigH*_LFH_up_fwd | GCGTGAGGATTTCCGTAATATGGAC |
| TM4176 | *sigH*_LFH_up_rev | CCTATCACCTCAAATGGTTCGCTGCTCGTCCTCCAACTGGCAAAAC |
| TM4177 | *sigH*_LFH_do_fwd | CGAGCGCCTACGAGGAATTTGTATCGGTGTGAAACGCAAGCTGGAGA |
| TM4178 | *sigH*_LFH_do_rev | GTTTCTTCTTCTTCGGGTACGACTAC |
| TM4986 | spaR_fwd | ATGCGAATTCGCGGCCGCTTCTAGAGATGGCAAAAATCCTTGCTGTCG |
| TM4987 | spaR_rev | ATGCCTGCAGCGGCCGCTACTAGTACTATTCCCATTTGTACCCAACG |
| TM6284 | spaIFEG_LFH_up_fwd | AGAGATGCTTGGTGTTATGG |
| TM6285 | spaIFEG_LFH_up_rev | CCTATCACCTCAAATGGTTCGCTGATTGTGTTCCTCCTTAAAGCC |
| TM6286 | spaIFEG_LFH_do_fwd | CGAGCGCCTACGAGGAATTTGTATCGGAAGAACAGGCAGGGAAAT |
| TM6287 | spaIFEG_LFH_do_rev | CATTCACTCTGGCACTAAGC |

^a^ E: *Eco*RI, P: *Pst*I, S: *Spe*I, X: *Xba*I.

^b^ Characters underlined: longer sequences: overhang for homologous regions of resistance cassettes, 6 nucleotide long sequences: recognition sites of restriction enzymes, single nucleotides: nucleotide exchange at this position.

References

1. Zeigler DR, Pragai Z, Rodriguez S, Chevreux B, Muffler A, Albert T, Bai R, Wyss M, Perkins JB. The origins of 168, W23, and other Bacillus subtilis legacy strains. J Bacteriol. 2008;190(21):6983-6995.

2. Popp PF, Dotzler M, Radeck J, Bartels J, Mascher T. The *Bacillus* BioBrick Box 2.0: expanding the genetic toolbox for the standardized work with *Bacillus subtilis*. Sci Rep. 2017;7(1):15058.

3. Radeck J, Kraft K, Bartels J, Cikovic T, Dürr F, Emenegger J, Kelterborn S, Sauer C, Fritz G, Gebhard S, Mascher T. The *Bacillus* BioBrick Box: generation and evaluation of essential genetic building blocks for standardized work with *Bacillus subtilis*. J Biol Eng. 2013;7(1):1-17.

4. Guérout-Fleury AM, Shazand K, Frandsen N, Stragier P. Antibiotic-resistance cassettes for *Bacillus subtilis*. Gene. 1995;167(1-2):335-336.
