# Supplementary figures and images for: Regulation of heterologous subtilin production in Bacillus subtilis W168
Source: Microb Cell Fact. 2022 Apr 7;21:57. doi: 10.1186/s12934-022-01782-9 (PMC8991943; doi:10.1186/s12934-022-01782-9)

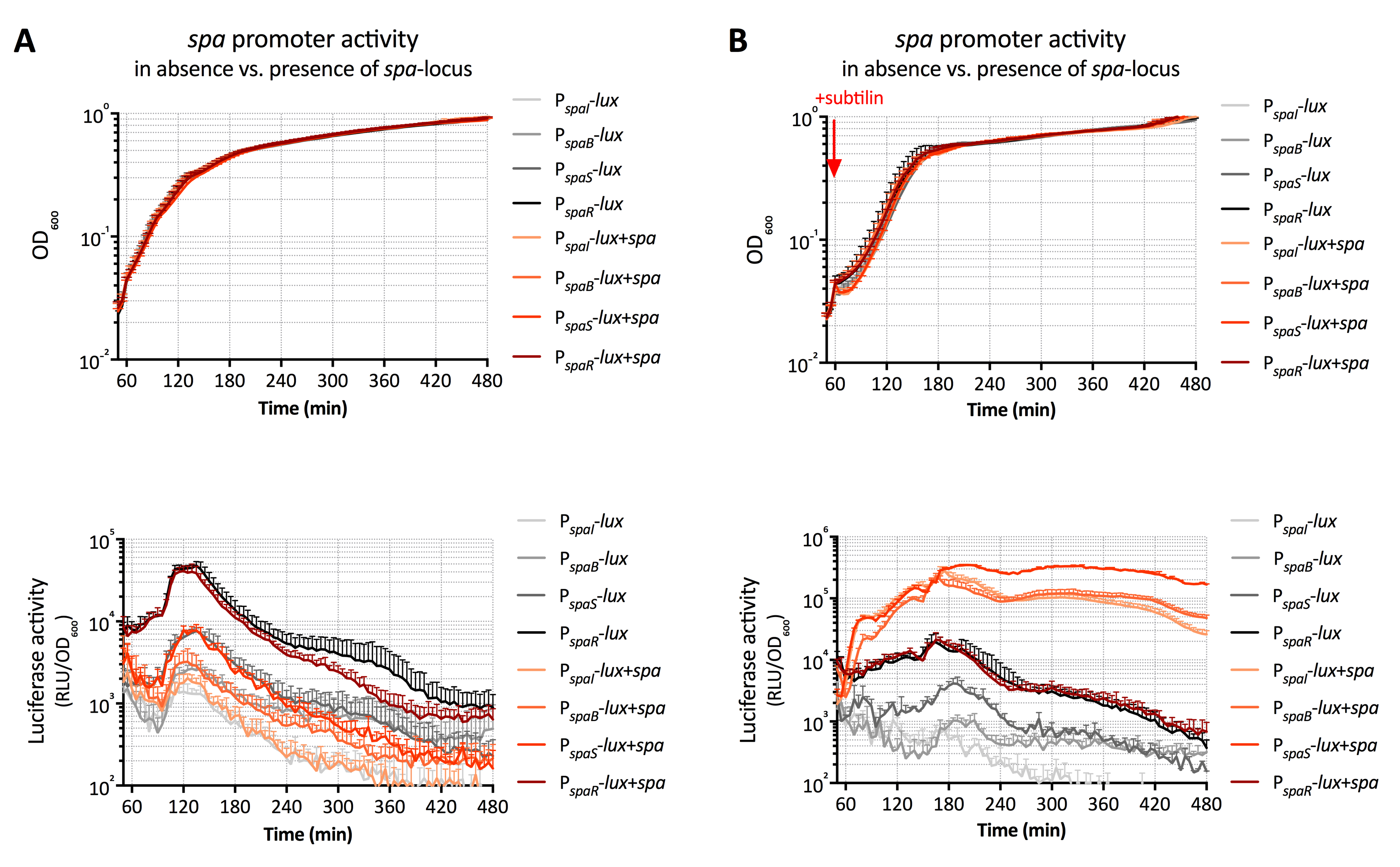

Supplement: Supplementary file 2 — Additional file 2: Fig. S1. Activity of spa promoters in absence and presence of spa-locus in B. subtilis W168. A represents the dynamics of spa promoter activity in the absence and presence of spa-locus in B. subtilis W168 along growth. B represents the response of spa promoters to the addition of extra subtilin supernatant in both conditions, showing the existing of spa-locus in the strain which caused the SpaRK-mediated autoregulation of subtilin biosynthesis. The color code of the strains is shown on the right side of the respective figure. Luciferase activity was defined as relative luminescence units (RLU) per OD600 (RLU/OD600). The mean values and standard deviations (error bars) of at least replicate measurements are shown. [file 12934_2022_1782_MOESM2_ESM.png]

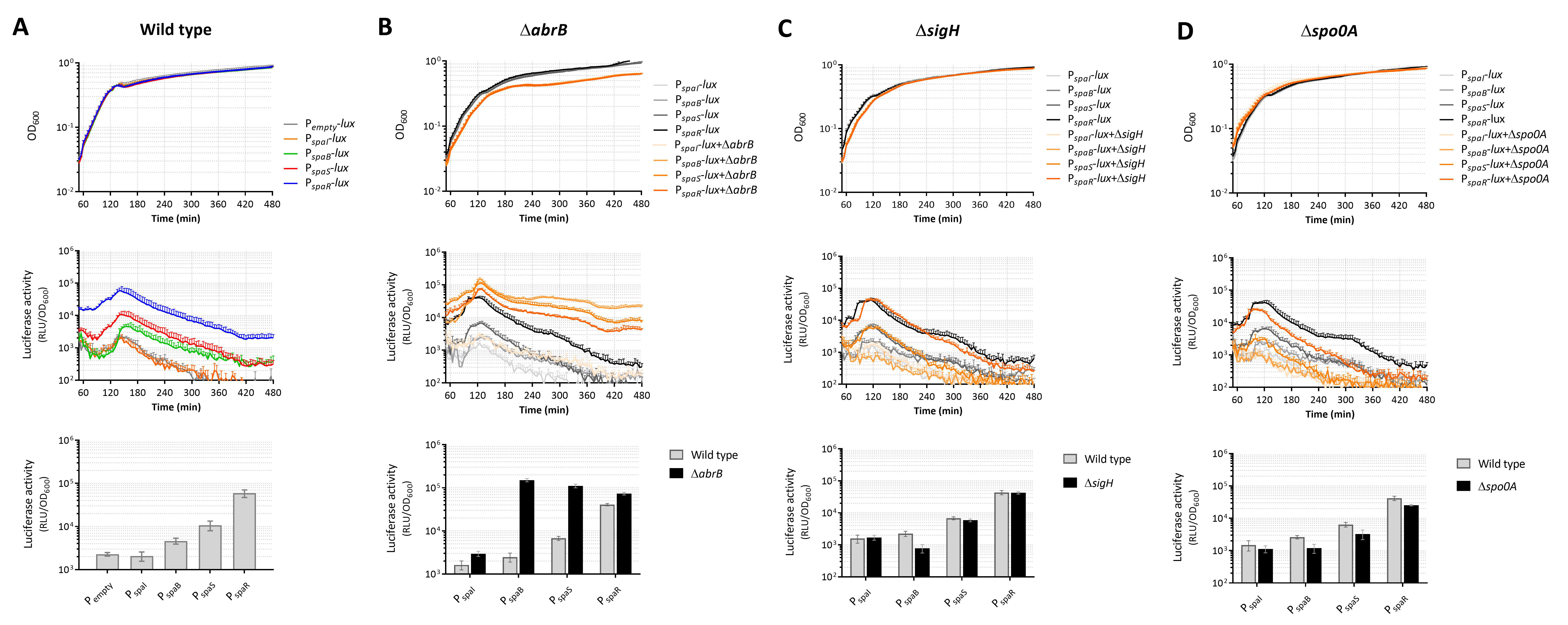

Supplement: Supplementary file 3 — Additional file 3: Fig. S2. Activity of spa promoters in wild type B. subtilis W168 and single mutants. A, B, C and D represent the dynamics of spa promoter activities in wild type B. subtilis W168, ΔabrB, ΔsigH, and Δspo0A mutants, respectively, in the absence of subtilin. Each of them contains the growth curves on the top, luminescence curves in the middle and the bar graph (as Fig. 2) at the bottom representing the peak luciferase activity shown in each strain. (The following description also applies to Fig. S3, Fig. S4 and Fig. S5) Luciferase activity was defined as relative luminescence units (RLU) per OD600 (RLU/OD600). The color code of each strain in each panel is depicted at the right side of the growth curves, respectively. The graphs show mean values and standard deviations (error bars) of at least triplicate measurements. [file 12934_2022_1782_MOESM3_ESM.jpg]

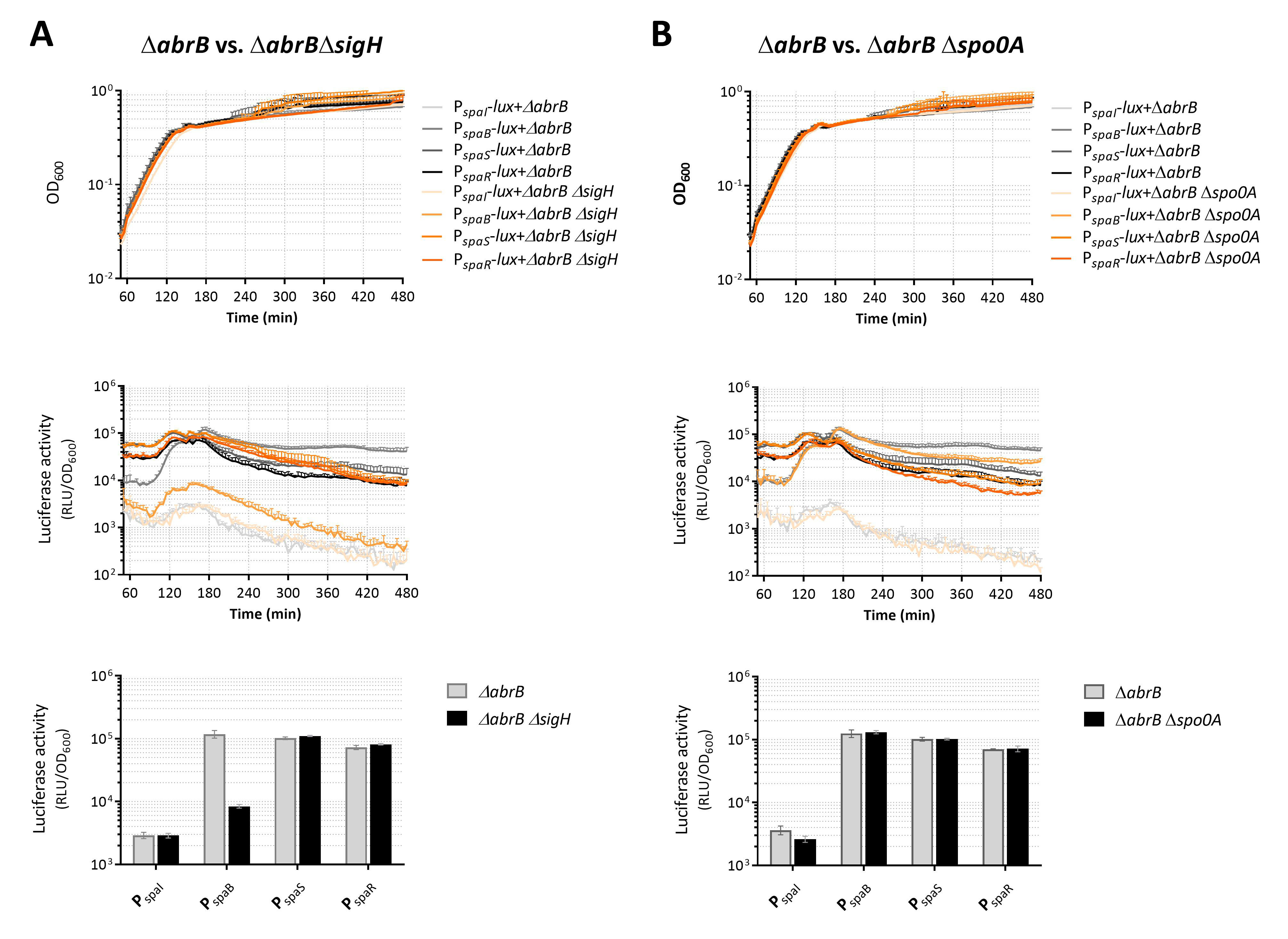

Supplement: Supplementary file 4 — Additional file 4: Fig. S3. Activity of spa promoters in B. subtilis W168 double mutants. A and B represent the dynamics of spa promoter activities in W168 ΔabrB ΔsigH and W168 ΔabrB Δspo0A double mutants, respectively, in the absence of subtilin. Each of them contains the growth curves on the top, luminescence curves in the middle and the bar graph (as Fig. 3) at the bottom representing the peak luciferase activity in each strain. [file 12934_2022_1782_MOESM4_ESM.jpg]

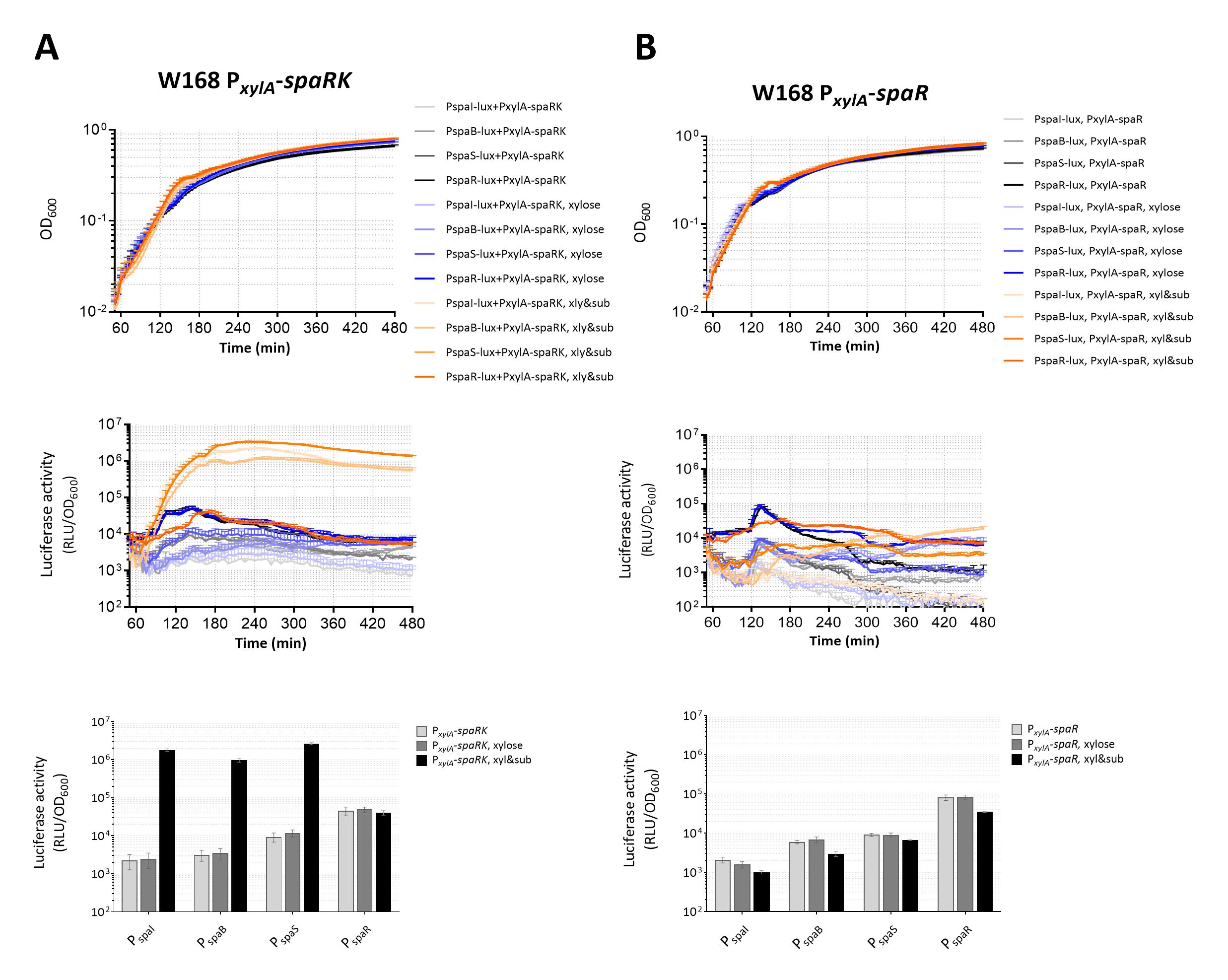

Supplement: Supplementary file 5 — Additional file 5: Fig. S4. Regulation of the two-component system (TCS) SpaRK on subtilin biosynthesis. A and B represent the dynamics of spa promoter activities in B. subtilis W168 PxylA-spaRK and B. subtilis W168 PxylA-spaR under different treatment conditions (untreated, treated with xylose and treated with both xylose and subtilin), respectively. PxylA indicates a xylose-inducible promoter. Each of them contains the growth curves on the top, luminescence curves in the middle and the bar graph (as Fig. 4) at the bottom representing the peak luciferase activity in each strain. [file 12934_2022_1782_MOESM5_ESM.jpg]

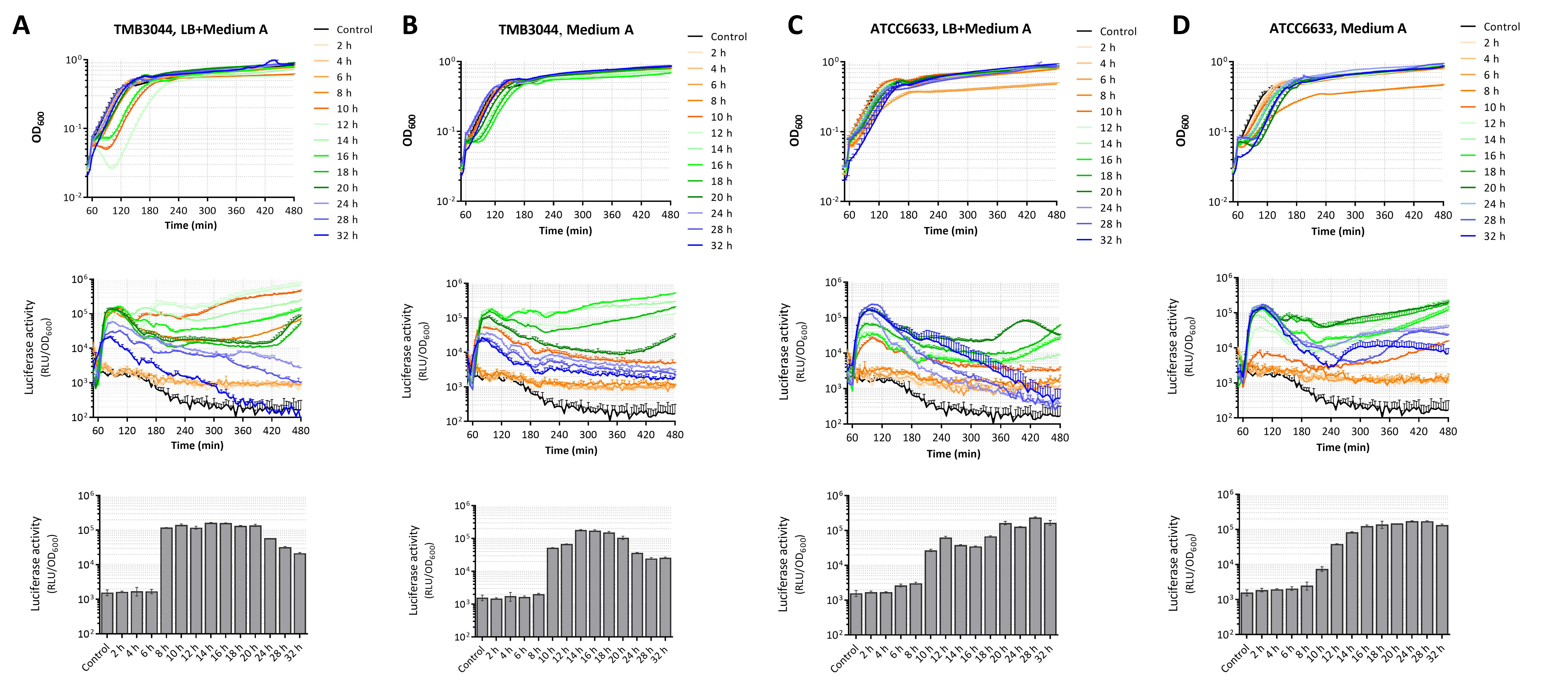

Supplement: Supplementary file 6 — Additional file 6: Fig. S5. Quantification of subtilin production. The subtilin supernatants collected from TMB3044 (W168 spa ΔabrB) and subtilin native producer B. subtilis ATCC6633 at defined time points of growth as indicated at the right side of the growth curves were tested using reporter strain TMB1617 (PliaI-lux). Mixed medium (50% LB + 50% Medium A) and Medium A were used for the cultivation, respectively, for both strains. The subtilin supernatant was added to the cell culture after 1 hour of growth in a microplate reader. Each of the A, B, C and D contains the growth curves on the top, luminescence curves in the middle and the bar graph (as Fig. 6) at the bottom representing the peak luciferase activity triggered by corresponding subtilin supernatant. [file 12934_2022_1782_MOESM6_ESM.jpg]

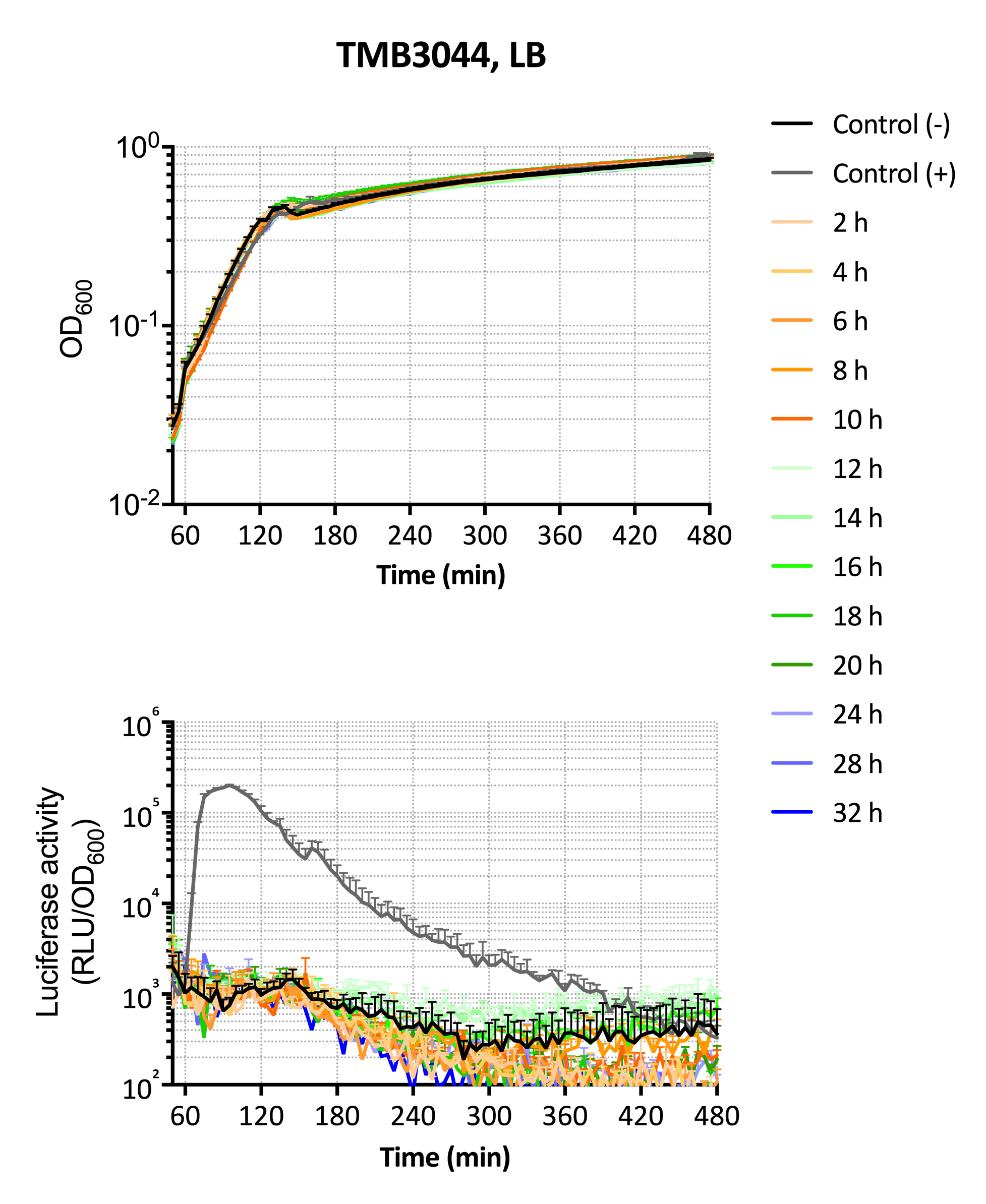

Supplement: Supplementary file 7 — Additional file 7: Fig. S6. Evaluation of subtilin production of TMB3044 cultured in LB medium. The subtilin supernatants collected at defined time points from TMB3044 (W168 spa ΔabrB) cultured in LB medium were tested using reporter strain TMB1617 (PliaI-lux). Control (-) indicates instead of subtilin supernatant the reporter strain was treated with the same volume of sterile water. Control (+) as the positive control was treated with 0.75% subtilin supernatant collected from B. subtilis ATCC6633 at the 20th hour of growth in Medium A. The upper panel shows the growth curves of the reporter stain under different treatments, and the bottom panel represents the luminescence curves under the corresponding treatments. The graphs show mean values and standard deviations (error bars) of three biological replicates [file 12934_2022_1782_MOESM7_ESM.png]

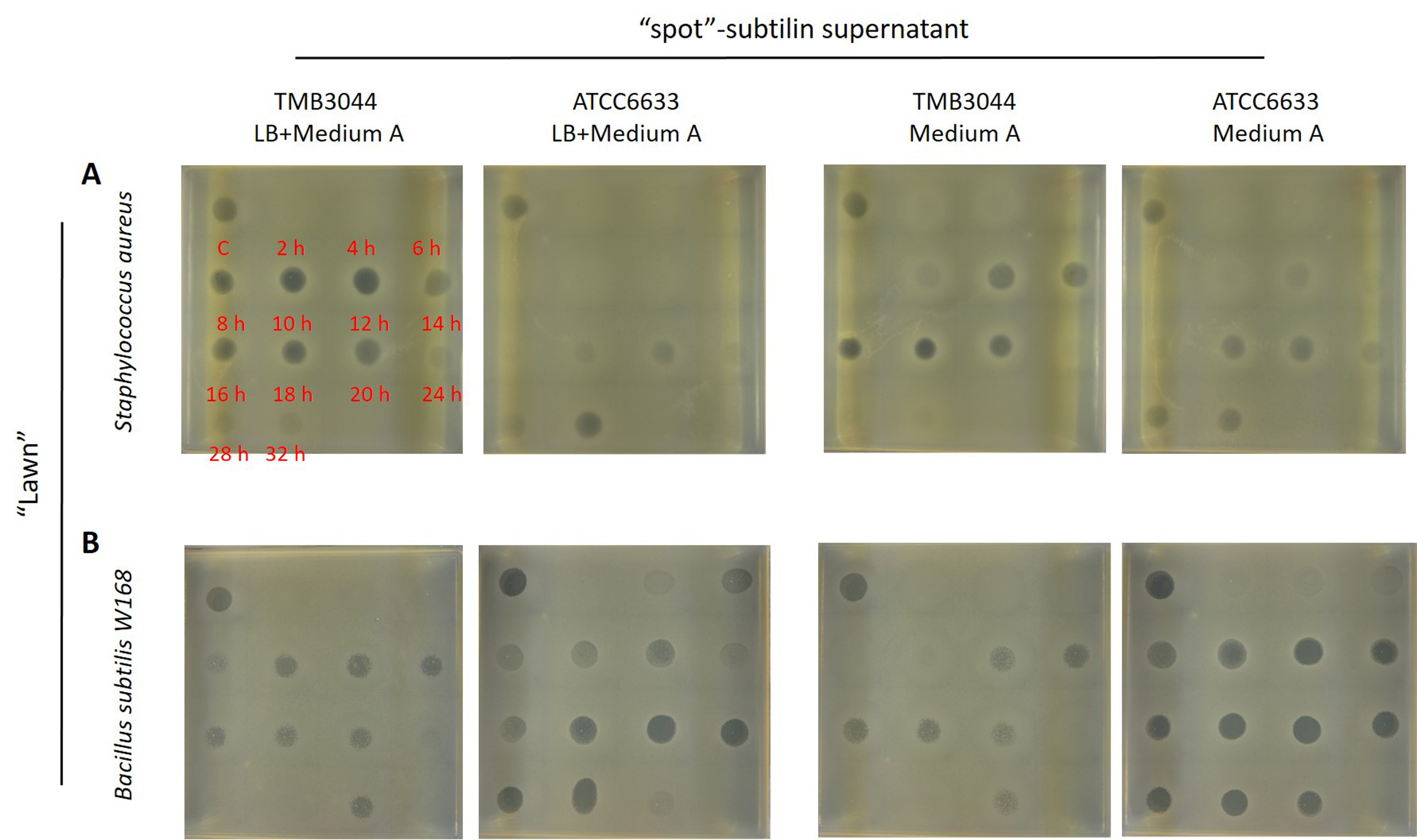

Supplement: Supplementary file 8 — Additional file 8: Fig. S7. Antimicrobial activity of subtilin against G+ bacteria S. aureus and B. subtilis W168. The same subtilin supernatant used in Fig. 6 were applied here, including the subtilin supernatant collected from TMB3044 (W168 spa ΔabrB) and B. subtilis ATCC6633 strain growing in mixed medium (50% LB + 50% Medium A), and 100% Medium A at defined time points as labeled on the top left plate in red. The control (indicated as C) was the subtilin supernatant collected from B. subtilis ATCC6633 at the 20th hour of growth in Medium A. [file 12934_2022_1782_MOESM8_ESM.jpg]

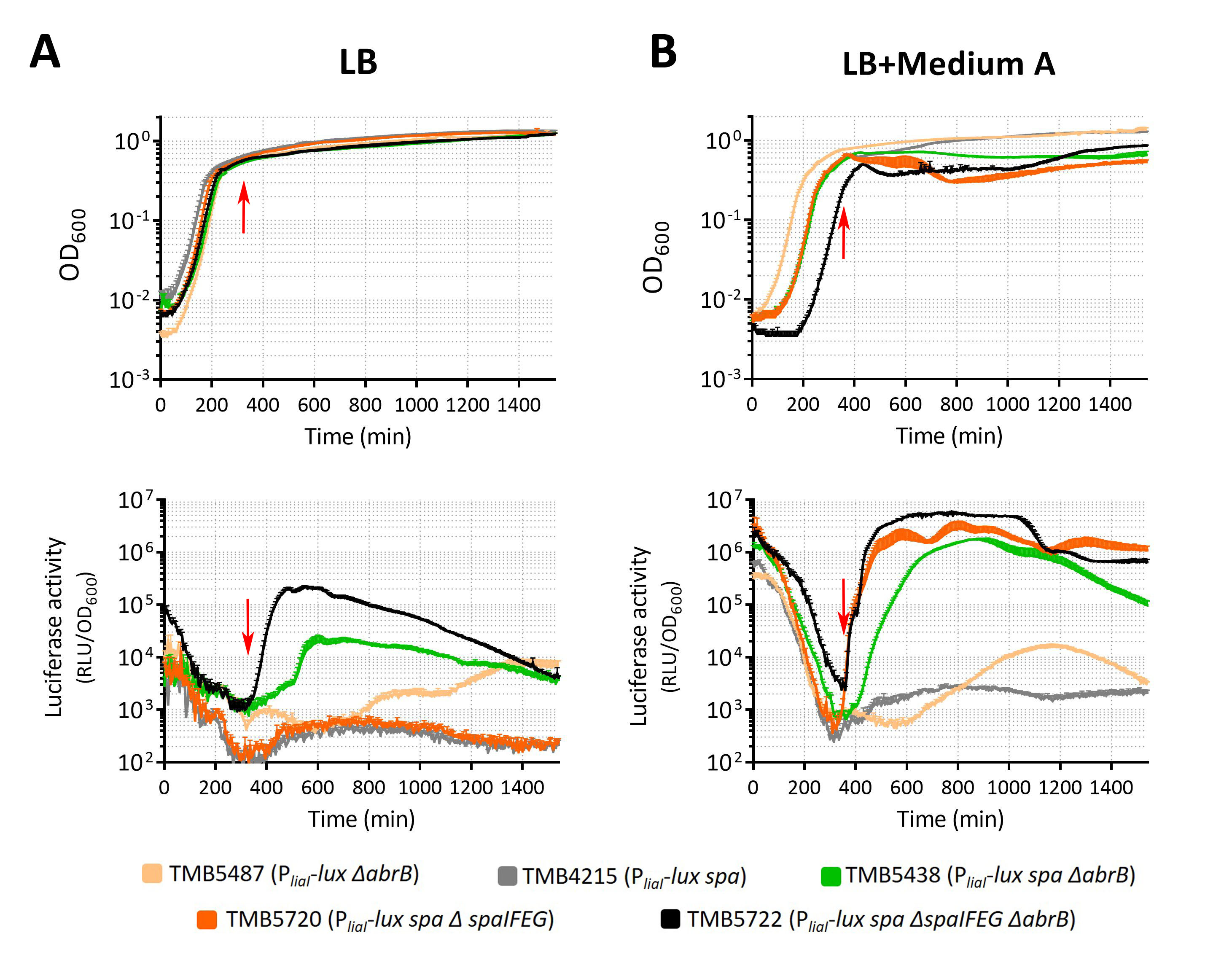

Supplement: Supplementary file 9 — Additional file 9: Fig. S8. “Online” monitoring the subtilin biosynthesis in B. subtilis W168 (PliaI-lux as the reporting system). The rationale of the response of PliaI to subtilin is given in Fig. 7A. “Online” reporters were examined in LB and mixed medium (50% LB + 50% Medium A), respectively. The genetic background and color-code of the strains are given at the bottom of the figure. The red arrows marked in luminescence graphs indicate the point where the induction starts in TMB5722 strain, while the corresponding arrow in the growth curve indicates the growth situation at the same time point. The mean of at least three replicates is shown with the error bars indicating the standard deviations. [file 12934_2022_1782_MOESM9_ESM.jpg]
